# The VBNC state: a fundamental survival strategy of *Acinetobacter baumannii*

**DOI:** 10.1128/mbio.02139-23

**Published:** 2023-09-28

**Authors:** Patricia König, Alexander Wilhelm, Christoph Schaudinn, Anja Poehlein, Rolf Daniel, Marek Widera, Beate Averhoff, Volker Müller

**Affiliations:** 1 Department of Molecular Microbiology & Bioenergetics, Institute of Molecular Biosciences, Goethe-University, Frankfurt am Main, Germany; 2 Institute for Medical Virology, University Hospital Frankfurt, Goethe University, Frankfurt am Main, Germany; 3 Advanced Light and Electron Microscopy ZBS4, Robert-Koch-Institute, Berlin, Germany; 4 Department of Genomic and Applied Microbiology & Göttingen Genomics Laboratory, Institute of Microbiology and Genetics, Georg-August University of Göttingen, Göttingen, Germany; LMU Munich, Munich, Germany

**Keywords:** pathogen, virulence, persistence, desiccation

## Abstract

The viable but non-culturable (VBNC) state is a persistence strategy adopted by bacteria to withstand long-lasting periods of unfavorable conditions. VBNC cells evade classical detection methods and are therefore easily transmitted in the hospital causing relapsing infections. The opportunistic human pathogen *Acinetobacter baumannii* has become a major threat in health care institutions and the food industry due to multiple antibiotic resistances and its ability to quickly adapt to very different ecological niches. Here, we report an additional, novel survival strategy of *A. baumannii*. Upon prolonged incubation in high-salt media, cells became unculturable. However, LIVE/DEAD staining followed by flow cytometry, respiratory activity assays, and resuscitation experiments revealed that these cells were viable but non-culturable. VBNC cells underwent large morphological changes. Entry into the VBNC state was also induced by pH and temperature stress, as well as by desiccation and anaerobiosis. The VBNC state was found in several strains of *A. baumannii*. Genome-wide expression profiling revealed a plethora of genes differentially regulated upon entry into the VBNC state. In summary, this study presents unequivocal evidence for a dormancy state in *A. baumannii* that has important consequences for detection of this pathogen and recurrent outbreaks.

**IMPORTANCE:**

Currently, the viable but non-culturable (VBNC) state is an underappreciated niche for pathogenic bacteria which provides a continuous source for recurrent infections and transmission. We propose the VBNC state to be a global persistence mechanism used by various *A. baumannii* strains to cope with many stresses it is confronted with in the clinical environment and in the host. This requires a novel strategy to detect viable cells of this pathogen that is not only based on plating assays.

## INTRODUCTION

*Acinetobacter baumannii* is a Gram-negative, opportunistic pathogen that has become a major threat in healthcare institutions worldwide. Due to its broad repertoire of antibiotic resistance mechanisms, the World Health Organization has put *A. baumannii* first place on the list of bacteria for which new therapeutics are urgently needed (1, 2). In addition, *A. baumannii* is extremely well adapted to the hospital environment and the human host: it can use different carbon and energy sources (3 – 5), it forms biofilms, and it is highly desiccation resistant and withstands high osmolarity (6, 7). The desiccation resistance is important for survival on abiotic surfaces whereas high osmolarity is encountered in the urine (8). The common biophysical feature of desiccation/osmolarity is a loss of free cellular water that is combated by *A. baumannii* by producing the compatible solutes glutamate, mannitol, and trehalose (9, 10). If the external salinity drops, mechanosensitive channels open to extrude solutes almost instantaneously, to avoid a massive water influx and cellular burst. But typically, the internal solute pool remains high as long as the outside salinity is high. However, when we followed the concentration of compatible solutes over time, we observed an unusual deprivation of compatible solutes in the beginning of the stationary phase, which led to a nearly complete loss of solutes within 10 hours, although the external salt concentration remained unchanged (11). Four days after entering, the stationary phase cells became unculturable but could be resuscitated by stress removal. This was reminiscent of a dormancy state called the viable but non-culturable state (VBNC) (12 – 14). Bacteria in the VBNC state are unculturable in medium that would normally support their growth, although they are alive and metabolically active, but with reduced rates (14, 15). These physiological changes are associated with large morphological changes (13). An important characteristic of the VBNC state is that non-culturable bacteria can be revived by stress removal (14, 15). VBNC bacteria not only have an increased stress tolerance including higher antimicrobial and antibiotic resistance but are even found to produce virulence factors during this state (15).

Whether or not *A. baumannii* uses the VBNC state is of critical importance to detect and combat this pathogen. Despite this fundamental importance, there is a controversy in the literature whether or not *A. baumannii* can enter the VBNC state. Early experiments did not support a VBNC state in *A. baumannii* (16), but our initial identification of VBNC in *A. baumannii* strain ATCC 19606^T^ was confirmed later by another group in strain AB-16 (11, 17). We have followed up these studies and will present evidence by a multitude of different methods that *A. baumannii* ATCC 19606^T^ enters the VBNC state as a response to prolonged low-water activities, ensuring its survival under these conditions for at least 10 months. More importantly, the VBNC state was found in different strains including clinical isolates, demonstrating this fundamental survival strategy in general in this species.

## RESULTS

### Evidence for a VBNC state in *Acinetobacter baumannii*

**Fig 1:**
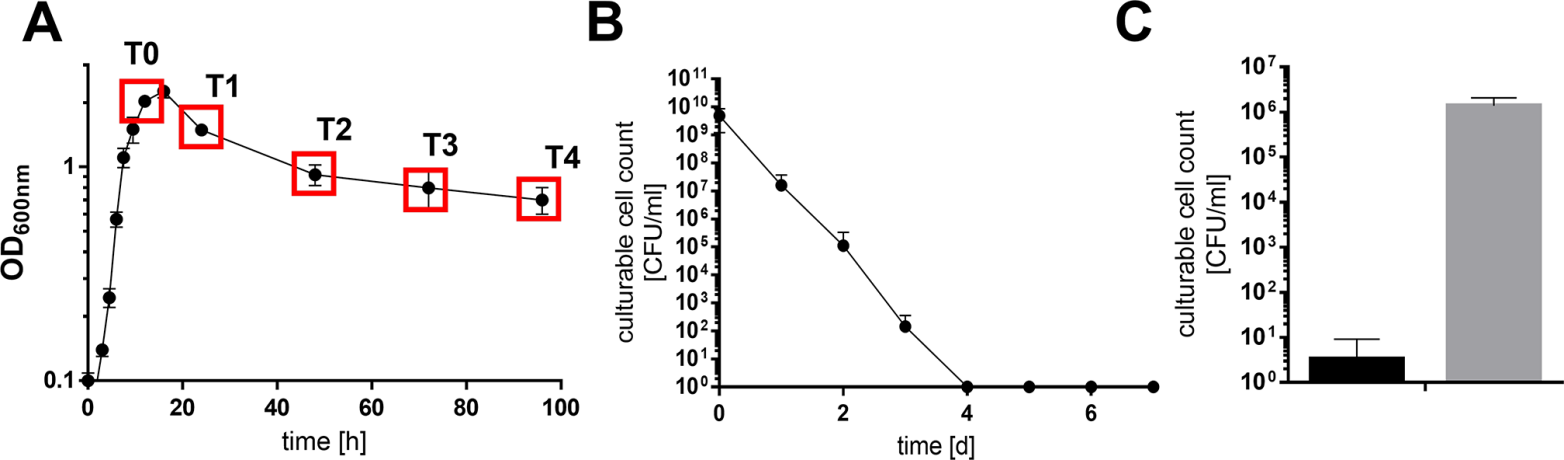
Salt-induced unculturability can be reversed by stress removal. *A. baumannii* ATCC 19606^T^ was grown in minimal medium with 20 mM succinate and 300 mM NaCl to the stationary growth phase (timepoint 0 [T0]) and incubated for further 4 days (**A**). At timepoints indicated (red squares), the culturable cell count (●) was determined by serially diluting the culture and plating samples onto LB agar plates (**B**). Once culturability dropped below 100 CFU/mL, a resuscitation assay was performed. Therefore, the stressor was removed by diluting cells 1:10 in sterile PBS. Samples were plated directly after inoculation (black bars) and after 2 days at 37°C (gray bars, **C**). Error bars denote the standard deviation of the mean derived from at least three independent biological replicates.

**Fig 2:**
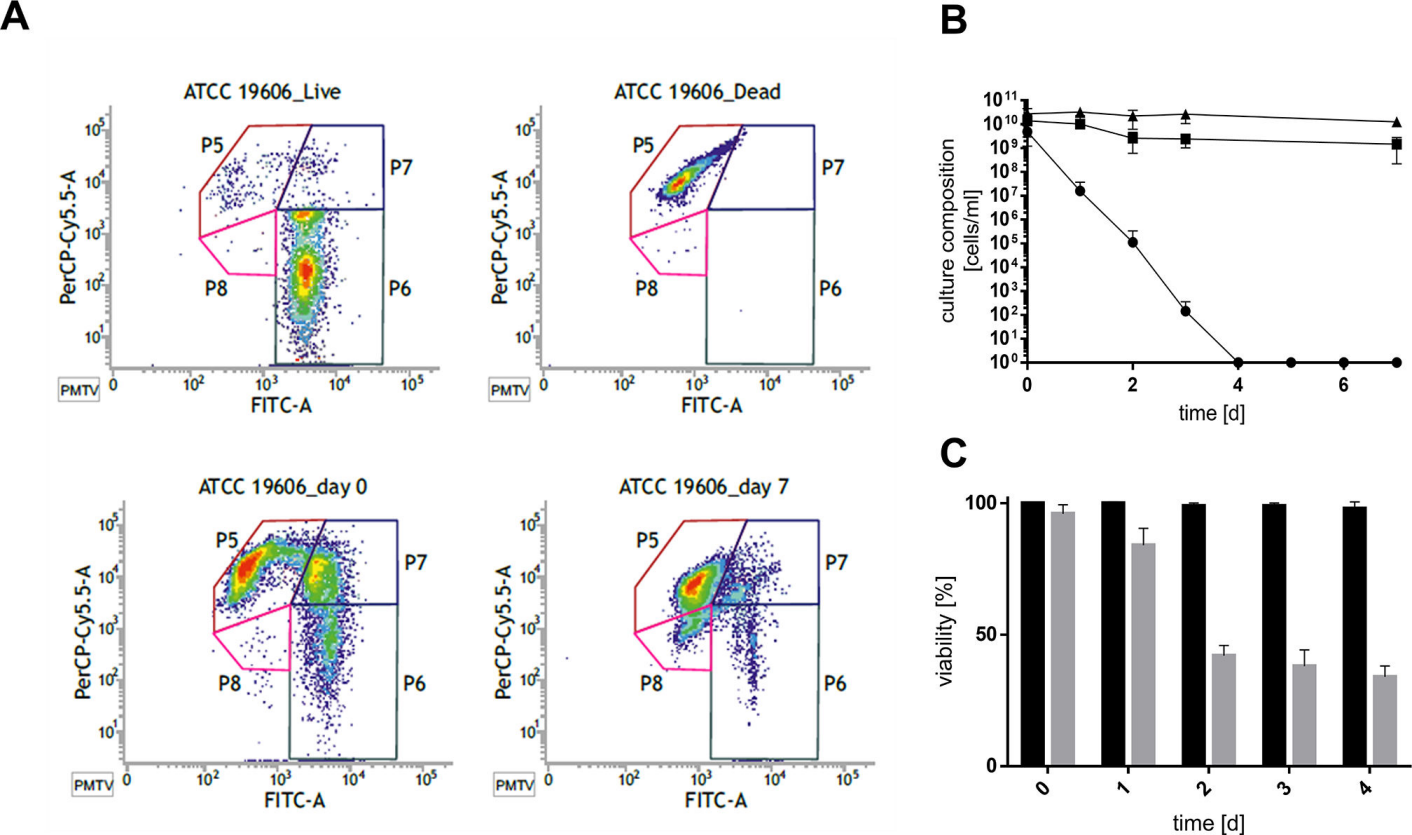
Survival under high-salt conditions as determined by LIVE/DEAD and CTC staining. *A. baumannii* ATCC 19606^T^ was grown in minimal medium with 20 mM succinate and 300 mM NaCl to the stationary growth phase (timepoint 0 [T0]) and for further 7 days. Cells were stained with the LIVE/DEAD BacLight Bacterial Viability Kit according to manufacturer’s instructions and analyzed by flow cytometry. Representative density plots from at least three independent biological replicates are shown (**A**). The viable cell count (■) is defined as the sum of populations 6 and 7, while the total cell count (▲) is defined as the sum of populations 5–8 and quantified using the kit’s microsphere standard. The culturable cell count (●) was determined by serially diluting the culture and plating samples onto LB agar plates (**B**). The respiratory activity of cells either grown in low- (black bars) or high-salt (gray bars) mineral medium was determined by using the BacLight RedoxSensor CTC Vitality Kit according to manufacturer’s instructions. Cells were counterstained with DAPI and visualized with CLSM prior to counting (**C**). Error bars denote the standard deviation of the mean derived from at least three independent biological replicates.

*A. baumannii* ATCC 19606^T^ was grown in the presence of high salt to the stationary growth phase and incubated further (Fig. 1A). Four days after entry into the stationary phase, the cell number detected by plating on LB agar plates decreased from 3.7 × 10^9^ CFU/mL to zero (Fig. 1B), as described before (11). When cells were incubated in resuscitation buffer (phosphate-buffered saline [PBS]) for 2 days and then plated, a high cell titer of about 1.4 × 10^7^ CFU/mL or 0.38% of the original count was restored, demonstrating that cells regained culturability after stress removal (Fig. 1C). This prompted us to analyze the physiological status of these cells in more detail. Therefore, cells were stained with Syto9 that stains living cells and with propidium iodide (PI) that stains dead cells only. Fluorescence microscopy revealed viable cells even after 4 days post stationary phase (PSP; Fig. S1). Flow cytometry was then used for quantification. As controls, culturable and isopropanol-inactivated cells were used (Fig. 2A). Analysis of the culture at day 0 PSP revealed a distinct dead (P5) and dominant viable population (P6). Further, a smaller double-stained population (P7) occurred, which is suggested to visualize damaged and living cells and was therefore added to the viable cell count. Forty-three percent of the stationary culture could thus be assigned as dead, but the majority was still viable. A comparison with the culture at four days PSP showed a clear decrease of viable cells. Nonetheless, 17.1% of the cells were found to be viable while the culturable cell count was zero. Interestingly, a so far unknown additional double-stained population (P8) occurred 4 days PSP which had 10.7% of the total cell count. In sum, the viable cell count remained constantly high in the 4 days PSP. The same was true for the total cell count (Fig. 2B).

Since Syto9/PI staining relies on membrane integrity and is prone to overestimate dead cells, we verified our finding using another staining procedure. This procedure relies on measuring the respiratory activity of cells using 5-cyano-2,3-ditolyl tetrazolium chloride (CTC). Actively respiring cells will import CTC and reduce it to insoluble formazan, which can be detected (Fig. S2). Cells were again grown with high salt, and their viability was addressed on 4 consecutive days PSP, while cells grown in low-salt medium served as control (Fig. 2C). Corresponding to our findings with the LIVE/DEAD staining, 96 ± 3.4% of the total cell count was found to be viable at day zero (T0) in the presence of high salt. The viable cell count declined steadily over the course of the experiment resulting in 34 ± 4.1% of viable cells 4 days PSP, although the cells were completely unculturable at this timepoint. Cells grown at low salt showed no significant decrease in viability 4 days PSP. These findings clearly supported our data of the LIVE/DEAD staining and are in line with the hypothesis that *A. baumannii* ATCC 19,606^T^ cells become non-culturable under high-salt conditions but are still viable, i.e., enter into a dormancy state.

### Unculturability can be reversed by stress removal within 10 months

**Fig 3:**
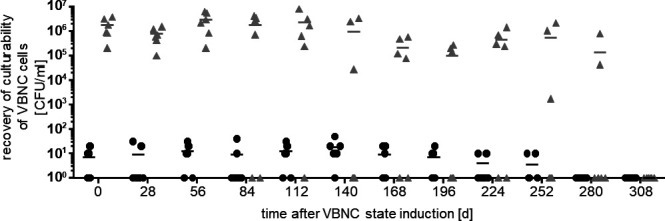
Recovery of culturability of VBNC cells. Unculturability of the strain ATCC 19606^T^ was induced by high-salt conditions. Resuscitation assays were performed monthly to address viability. The stressor was removed by diluting cells 1:10 in sterile PBS. Samples were plated directly after transfer (black bars) and after 2 days at 37°C (gray bars). Each dot represents a biological replicate, and the mean of six independent experiments is given by the black line.

To address the timeframe in which cells can be revived from the VBNC state, a VBNC culture was further incubated for 11 months PSP and the resuscitation assay was repeated monthly (Fig. 3). Within the first 3 months, resuscitation efficiency remained stable at about 1.8 × 10^6^ CFU/mL before single samples failed to regain culturability. After 7 months, the resuscitation efficiency started to fluctuate strongly between 2.1 × 10^6^ and 1.7 × 10^3^ CFU/mL with a success rate of only 30–50%. Eleven months after entry into the VBNC state, none of the samples could be resuscitated after repeated attempts of stress removal by dilution in PBS. The addition of succinate to PBS did also not lead to successful resuscitation. However, when resuscitation was performed in LB medium, cells started to grow after approximately 48 hours (3.1 × 10^8^ CFU/mL), indicating the presence of a component in LB that supports resuscitation.

### The cell morphology changes in VBNC cells

**Fig 4:**
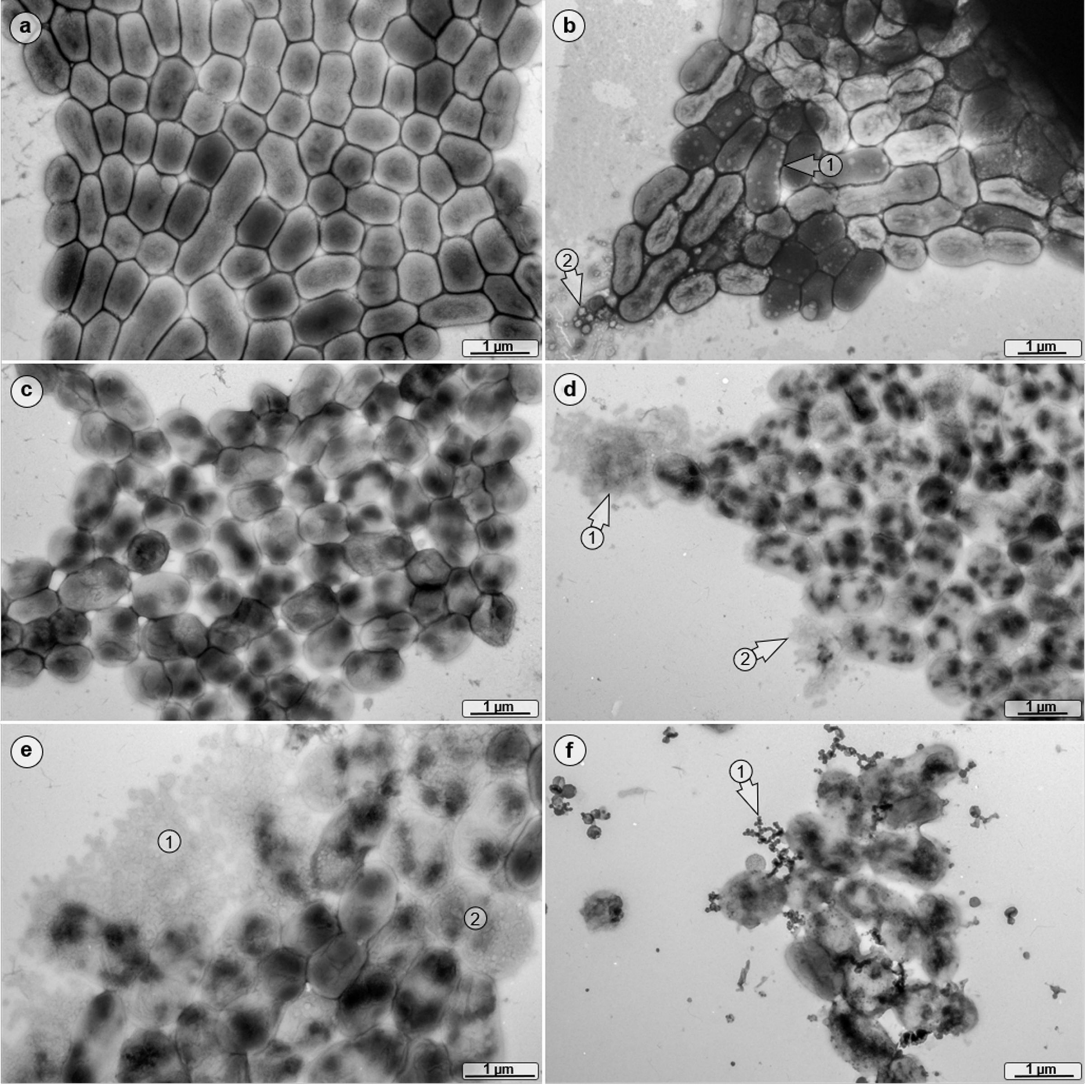
Morphological analyses of VBNC cells. ATCC 19606^T^ was grown in either low- (**a**) or high-salt (**b**) mineral medium, and TEM images were obtained at T0 and 4 days PSP. High-salt-treated cells produced numerous intracellular vesicles (arrow 1), and extracellular cell debris was detected also (arrow 2). (**c**) The cells of the low-salt culture 4 days PSP exhibited an altered cell morphology. (**d**) In the high-salt culture 4 days PSP, cytoplasm-leaking bacteria were detected (arrows 1 and 2) and (**e**) a high percentage of disintegrated bacterial cells was present (areas 1 and 2). (**f**) Resuscitated cells formed many short dendritic, globular strands as an integral part of bacterial clusters (arrow 1).

To analyze possible morphological changes of VBNC cells, transmission electron microscopy was used (Fig. 4). Cells of *A. baumannii* ATCC 19606^T^ grown at low salt exhibited a nearly rod shape structure with a length of 0.85 × 0.94 µm (Fig. 4a). VBNC cells had a different morphology, 73.1% of the VBNC population contained numerous intracellular vesicles, and first signs of extracellular debris were detected while this was the case in only 0.7% of the culturable cell population (Fig. 4b). Four days PSP in low-salt media, the staining pattern of 0.3% of the culturable *A. baumannii* cells indicated a non-homogeneous distribution of the cytoplasmic content (Fig. 4c). The latter effect was even more pronounced in VBNC cells and present in 88.5% of the population (Fig. 4d) and was accompanied by a high percentage of disintegrated bacteria (20.6%; Fig. 4d and e). Furthermore, some of the VBNC cells exhibited a rounder shape in comparison with non-VBNC cells. After resuscitation, the uneven staining pattern was still observed, as well as many short globular strands in their direct vicinity (Fig. 4f).

### Genome-wide expression profiling in VBNC state cells

**Fig 5:**
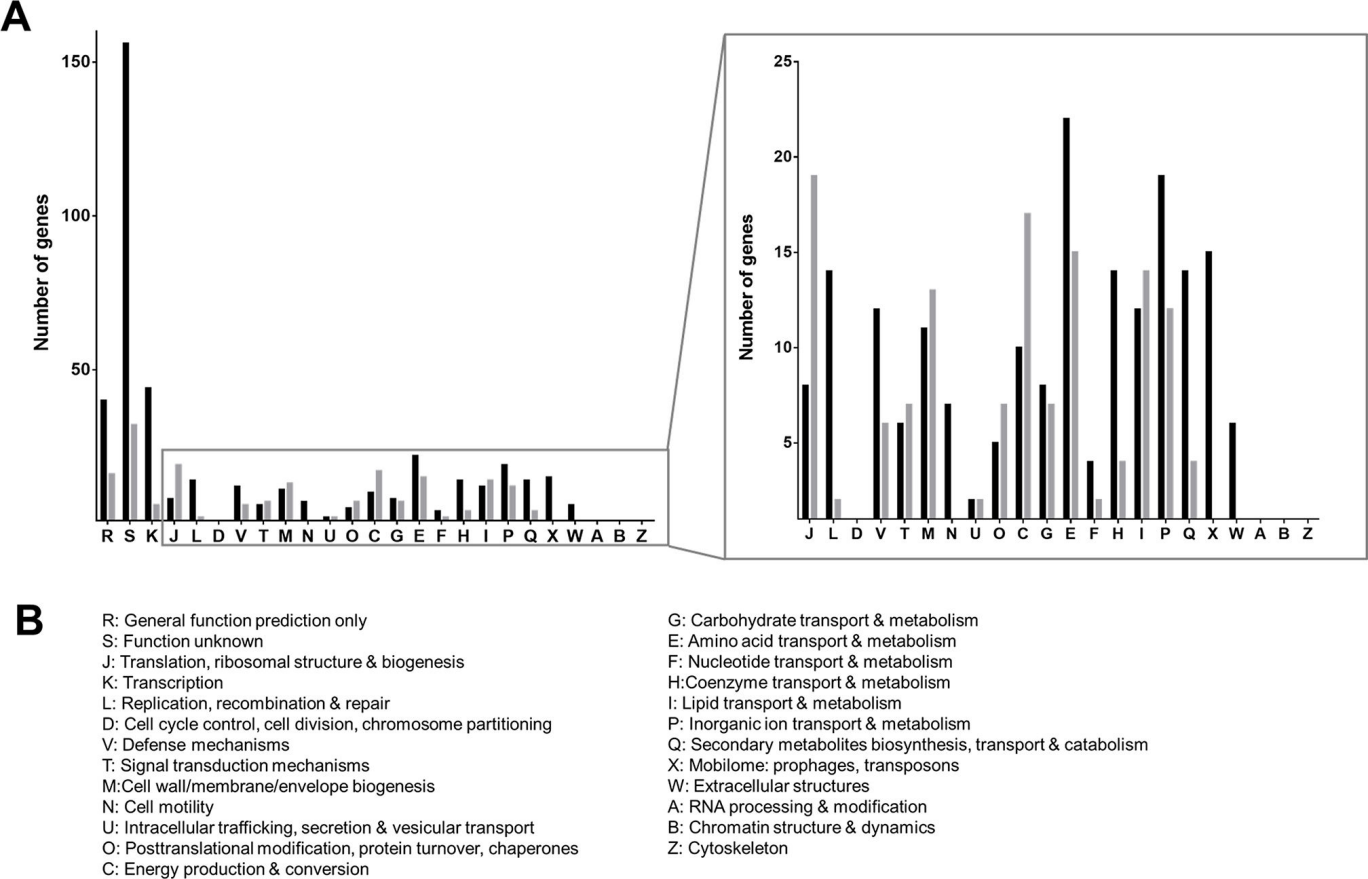
Differentially expressed genes of VBNC cells. (**A**) Upregulated (black bars) and downregulated (gray bars) genes (*P* ≤ 0.05 and a log_2_FC ≥ 2) were divided into functional groups in alignment with the COG category database which are listed in panel **B**.

Knowledge about the processes involved in VBNC-state induction in general is scarce (15). Therefore, we examined differences in gene expression levels in cells at day zero compared with 4 days PSP by genome-wide expression profiling. We identified 618 differentially expressed genes (DEG) from which 432 genes were up- and 186 genes were downregulated in the VBNC state (Fig. S3). A complete list of DEGs is given in Table S1. The majority of upregulated genes (25.6%) was from the category “function unknown” and “general function prediction only” (6.6%) (Fig. 5); 7.2% were from the category “transcription” and 2.3% from “replication, recombination, and repair,” including 38 transcriptional regulators, suggesting massive regulatory processes within the VBNC state as well as the upregulation of DNA repair mechanisms. The remaining DEGs are split quite evenly between the different metabolic routes (Fig. 5). DEGs with potential importance for entry into the VBNC state are described below.

### DEGs involved in metabolism

The majority of genes responsible for central carbon metabolism and energy conversion was downregulated in VBNC-state cells (Fig. S4A), including tricarboxylic acid cycle (TCA) genes suggesting a slowdown of the entire metabolic machinery. This is in line with the observation that many genes coding for enzymes that catalyze critical steps of different degradation/catabolic pathways were downregulated (e.g., pyruvate metabolism). In contrast, expression of genes of the respiratory chain was only slightly affected. In general, expression levels of transport systems such as the MFS transporter for uptake of nutrients/carbon and energy sources were extremely high (Fig. S4B). These analyses are in line with the hypothesis that VBNC cells are energetically compromised.

### DEGs involved in general stress adaptation

Since VBNC cells are known for their extraordinary resistance against antibiotics and general stressors (18), we checked for genes involved in these resistance mechanisms (Fig. S4C). Several drug efflux systems (RND and ABC transporter), as well as glutathione S-transferases, were highly upregulated, suggesting export and detoxification processes to occur. Strikingly, proteases and a cold shock protein were repressed, indicating a defect in protein degradation in VBNC-state cells, which is important for recycling processes and cell protection. Among the stress resistance-related genes, a recently recognized intrinsically disordered protein (HTZ92_1883) stood out. This protein was described to be essential for desiccation resistance in *A. baumannii* AB5075 and named DtpA for its desiccation tolerance-promoting property (19, 20). The majority of genes involved in the oxidative stress response was downregulated, while genes annotated as “endonucleases” were highly upregulated. Interestingly, two dihydrodipicolinate synthases were upregulated under VBNC-state conditions. They are involved not only in lysine biosynthesis but also in metal chelator production, indicating an increased need of metal ions in this state.

### DEGs involved in virulence associated traits

**Fig 6:**
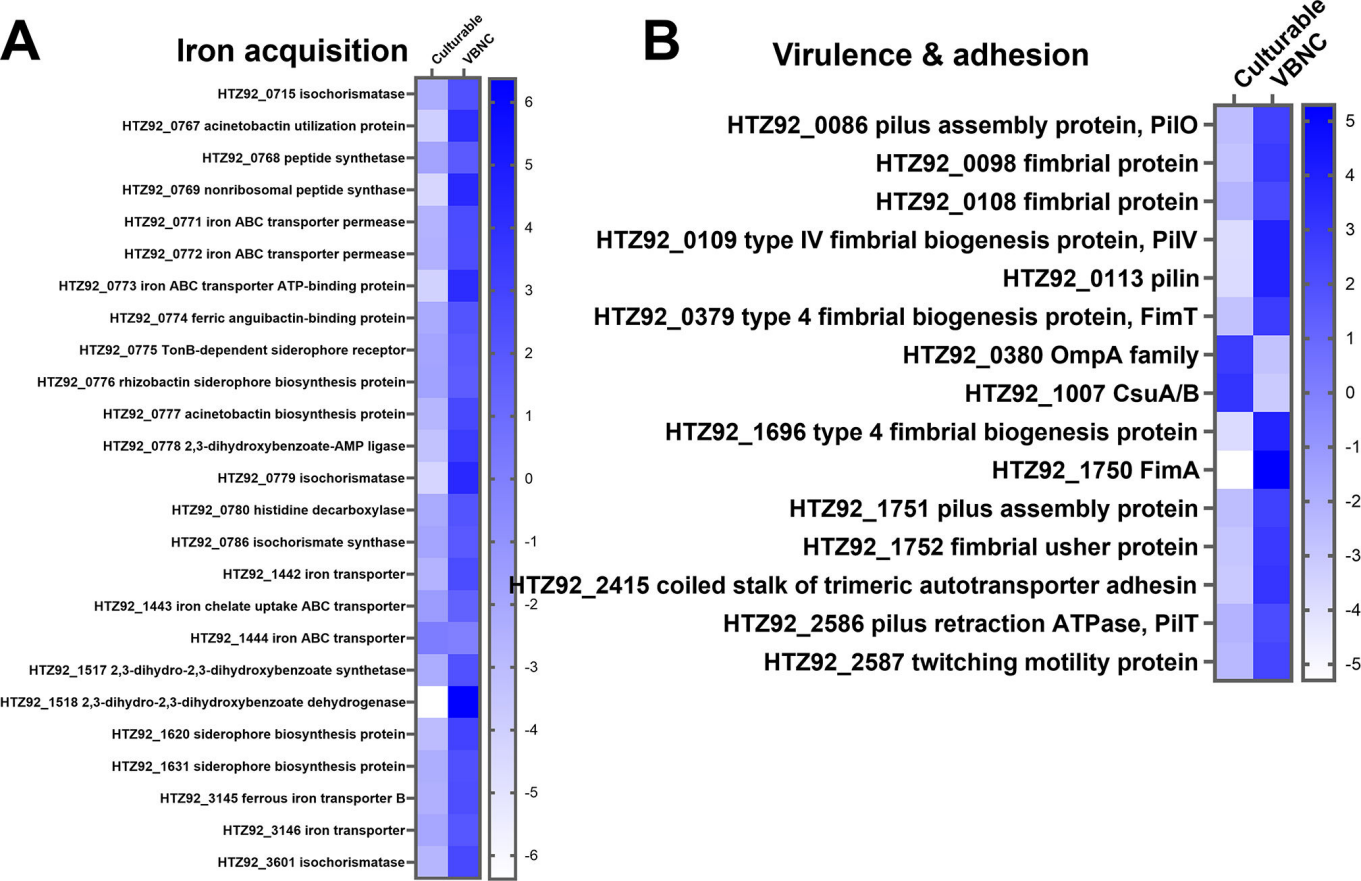
Top-ranked differentially regulated genes in the categories iron acquisition (**A**) and virulence & adhesion (**B**). Heatmaps of up- and downregulated genes (*P* ≤ 0.05 and a log_2_FC ≥ 2) involved in iron acquisition and virulence & adhesion. The log_2_FC (ranging from −6 to +6) is visualized with a color code represented by the color bar on the right. Figures were generated with GraphPad Prism 9.

One important feature of bacterial pathogenesis is the ability for efficient iron acquisition. *A. baumannii* VBNC cells showed an intensive upregulation of genes involved in iron scavenging processes (Fig. 6A). This included all iron uptake systems. Furthermore, biosynthesis routes for the siderophores acinetobactin, acinetoferrin, and anguibactin were upregulated, as well as genes encoding for all known iron uptake systems (Fig. 6A). Interestingly, the DEGs involved in the COG category “cell motility” were also highly upregulated in VBNC cells, including several genes responsible for virulence-related adhesion processes, including *fimA*, which belongs to the top-ranked upregulated genes in VBNC-state cells. In contrast, the characteristic virulence factors *ompA* and *csuA/B* were strongly downregulated (Fig. 6B).

### VBNC-state entry is C-source dependent

To analyze the effect of different C-sources on the VBNC-state entry, cells were grown on either 20 mM glutamate, 10 mM histidine, 20 mM alanine, 20 mM xylose, 20 mM arabinose, and 20 mM ethanol in the presence of 300 mM NaCl (Fig. S5A and S5B). All cultures became unculturable, and the timeframe in which culturable cells could still be detected (further referred to as “cultivation interval”) ranged from 7 to 63 days depending on the carbon source used. Cells could be resuscitated by stress removal, indicating VBNC-state entry under all conditions tested (Fig. S5C and D).

Since succinate oxidation increases the medium pH up to pH 9.5, we addressed the role of pH in VBNC-state induction and its carbon-source dependence. First, we checked the pH of the medium after growth with the carbon sources tested above, and strikingly, the corresponding cultivation intervals correlated strongly with the pH: the more alkaline the medium, the earlier the VBNC state was induced (Table S2). When cells were grown on arabinose (no pH change during growth) in the presence of 300 mM NaCl and pH 10, cells became unculturable within 3 days of incubation, similar to cells grown on succinate in the presence of high salt (Fig. S6). Not only high-NaCl concentrations but also 300 mM KCl, 300 mM Na^+^-gluconate, 600 mM sucrose, or 600 mM glucose induced the VBNC state, demonstrating high osmolarity in general as inducer of the VBNC state (Fig. S7).

### VBNC state is induced by different stressors

**Fig 7:**
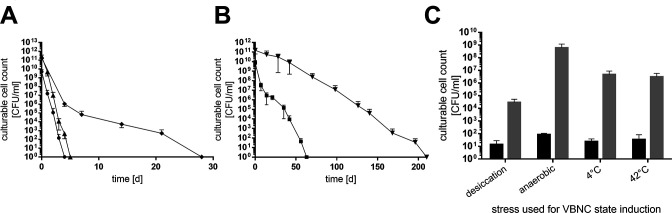
Different environmental stresses induce the VBNC state. (**A**) ATCC 19606^T^ was incubated under high salt (●), anaerobiosis (▲), and heat (♦, 42°C) stress as well as (**B**) desiccation (■) and cold stress (▼). Cultivation conditions were described in Material and Methods. Culturability was determined by plating on LB plates at timepoints given. (**C**) Once culturability dropped below 100 CFU/mL, a resuscitation assay was performed. The stressor was removed by diluting cells 1:10 in sterile PBS. CFUs were determined directly after transfer (black bars) and after 2 days at 37°C (grey bars, **C**). Error bars denote the standard deviation of the mean derived from at least three independent biological replicates.

Next, we addressed whether the entry into the VBNC state is also induced by other stressors. Indeed, desiccation, anaerobiosis, cold (4°C), and heat (42°C) stress also induced the entry into the VBNC state (Fig. 7). However, the timepoint of unculturability varied strongly depending on the inducer used, ranging from 6 ± 1 day (anaerobiosis) to 210 ± 21 days (4°C), while desiccated and heat-stressed cells were culturable for up to 63 ± 7 and 28 ± 4 days, respectively (Fig. 7A and B). Although the cultivation interval was inducer dependent, successful resuscitation was not (Fig. 7C). Culturability of *A. baumannii* ATCC 19606^T^ could be regained in each case, underlining that VBNC-state entry is a common mechanism for coping with prolonged stress adaptation. However, it is noteworthy that the resuscitation efficiency varied strongly. Desiccated cells lost major parts of their population, such as only 2.9 × 10^4^ CFU/mL were detected after resuscitation, whereas after heat and cold stress 4.6 × 10^6^ and 3 × 10^6^ CFU/mL were detected after resuscitation, respectively. Highest numbers of resuscitated cells were observed after anaerobiosis (5.9 × 10^8^ CFU/mL).

### Temperature modulates the timepoint of VBNC-state entry

After having seen the accelerating effect of additional pH stress on the timepoint of VBNC-state entry, we addressed the influence of different temperatures on the VBNC-state induction of previously stressed cells. Therefore, cells were grown on succinate in the presence of high sodium chloride before they were exposed to 4, 37, and 42°C, respectively (Fig. S8). As observed before, salt-stressed cells at 37°C became unculturable within 4 days PSP, but at 4°C, no decline in the culturable cell count could be observed 4 days PSP. In contrast, cells exposed to 42°C were already unculturable after 2 days PSP. These results demonstrate that the entry into the VBNC state can be induced by two different stressors but the response can be amplified or reduced.

### Entry into the VBNC state is a general feature among *A. baumannii* strains

**Fig 8:**
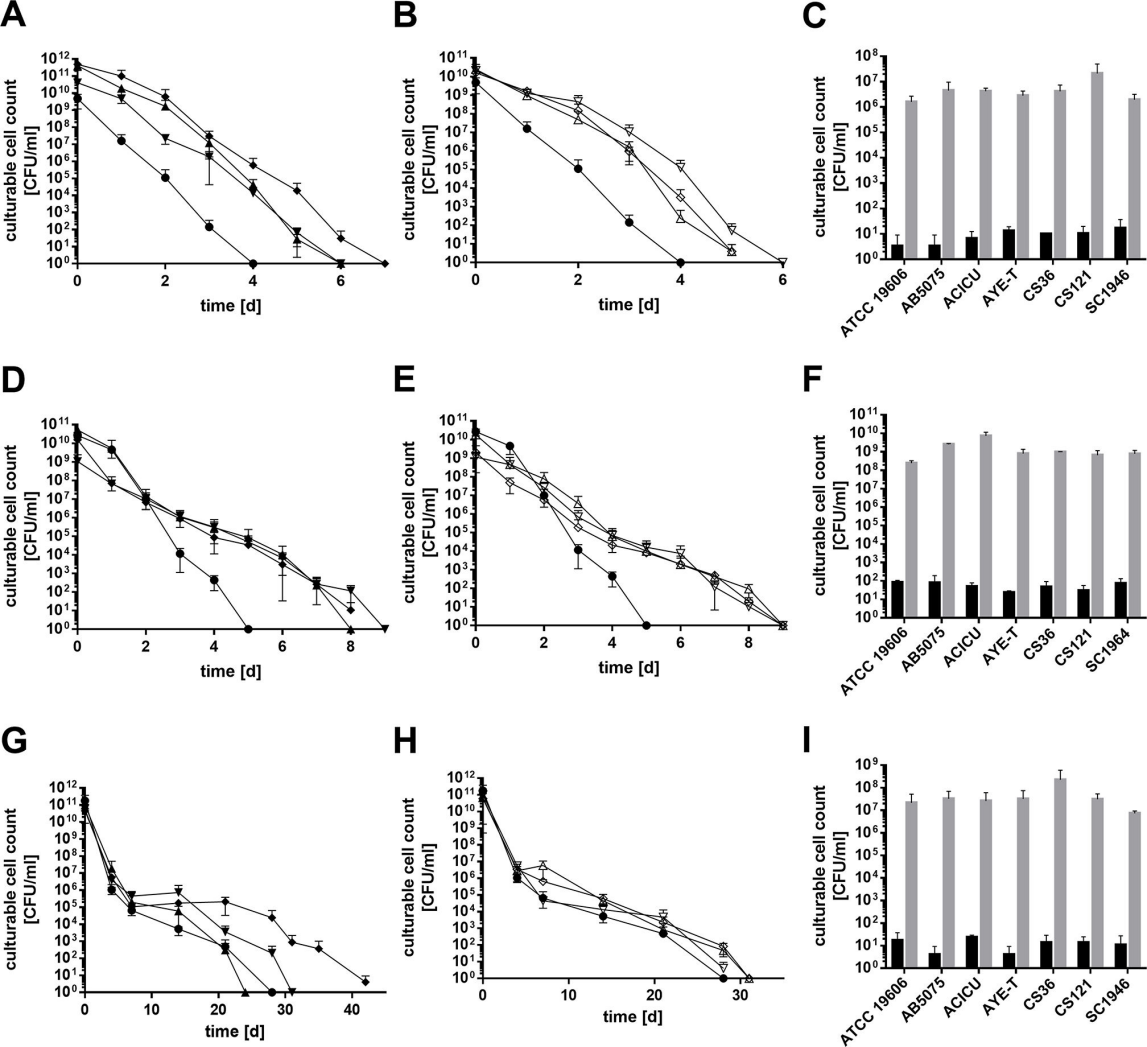
Different environmental stresses induce the VBNC state of clinically relevant strains. The number of culturable cells of ATCC 19606^T^ (●), AB5075 (▼), AYE-T (♦), ACICU (▲), CS36 (♢), CS121 (∇), and SC1947 (∆) incubated under high salt (**A and B**), anaerobiosis (**D and E**) and heat stress was determined (**G and H**). For comparison, ATCC 19606^T^ (●) is included in every graph. Cultivation conditions were described in Material and Methods. Culturability was determined by plating on LB plates at timepoints given (**C, F, and I**). Once culturability dropped below 100 CFU/mL, a resuscitation assay was performed. The stressor was removed by diluting cells 1:10 in sterile PBS. CFUs were determined directly after inoculation (black bars) and after 2 days at 37°C (gray bars, **C**). Error bars denote the standard deviation of the mean derived from at least three independent biological replicates.

The more virulent strain AB5075 also became unculturable in high-NaCl medium within 6 days PSP and also could be resuscitated (4.4 × 10^6^ CFU/mL; Fig. S9A and S9B). Viability analyses by flow cytometry provided clear evidence that also strain AB5075 enters the VBNC state in the presence of prolonged salt stress (Fig. S9C and S9D). The same was observed for the strains AYE-T, ACICU, CS36, CS121, and SC1946 (Fig. 8). High-NaCl concentrations led to a fast decrease of culturability of all strains tested, with ATCC 19606^T^ being the least (4 days of culturability) and AYE-T being the most resilient (7 days of culturability, Fig. 8A and B); all cultures could be resuscitated with efficiencies ranging from 1.61 × 10^6^ to 2.11 × 10^7^ CFU/mL (Fig. 8C). All strains except for ATCC 19606^T^ were culturable for up to 9 days under anaerobiosis, while ATCC 19606^T^ could not be cultivated 5 days PSP (Fig. 8D and E). Again, the resuscitation assay proved VBNC-state entry of all strains (Fig. 8F). High temperature (42°C) affected the long-term culturability of the strains quite differently (Fig. 8G). Interestingly, there was no difference in long-term culturability of ATCC 19606^T^ and the clinical isolates at 42°C (Fig. 8H). Again, all strains could be resuscitated (7.37 × 10^6^ to 2.24 × 10^8^ CFU/mL after resuscitation [Fig. 8I]). Cold stress increased the cultivation interval. ATCC 19606^T^ and CS36 were culturable for 210 and 196 days, while the other strains were even culturable after 294 days (Fig. S10A and S10B). Under desiccation conditions, ATCC 19606^T^ lost culturability after 63 days (Fig. S10C), while the more virulent reference strain ACICU was culturable for at least 147 days. The other strains were culturable throughout the whole incubation time (Fig. S10D). In fact, their culturable cell count declined very slowly and reached a final cell count of 2.79 × 10^6^ to 5.7 × 10^7^ CFU/mL by the end of the experiment.

## DISCUSSION

Here, we demonstrated that several *A. baumannii* strains became unculturable after a few days under high-salt conditions and succinate as carbon source. LIVE/DEAD staining, CTC measurements, and successful resuscitation analyses provide unequivocal evidence that *A. baumannii* enters the VBNC state. Successful resuscitation of non-culturable cells is seen as the most vital proof for the VBNC state; however, the specific conditions needed to regain culturability depend on the species under study, the applied stress condition, and the resuscitation time window (13, 15, 21, 22). Moreover, many cases are known in which VBNC cells cannot be resuscitated (21). The simplest approach is stress removal, which was applied in this study and led to a successful resuscitation of 1.8 × 10^−3^% to 36.9% of the viable population which is in the same range as in other bacteria (23 – 25). In our case, cells could be revived independently of the stressor and strain used. Under high-salt (NaCl) conditions, ATCC 19606^T^ could be resuscitated for up to 10 months and the experiment is still ongoing. The size of the resuscitation window varies strongly between different species, like 3 days in *Vibrio vulnificus*, 10 days in *Escherichia coli*, or even 11 years in the case of *Citrobacter freundii* (22, 26, 27). Here, after nearly 1 year of incubation at high-NaCl concentrations, successful resuscitation could still be accomplished by changing the resuscitation medium to LB. Since resuscitation was not possible by the addition of succinate to PBS but by the addition of LB medium, it can be assumed that LB contains components that support resuscitation. A similar phenomenon was described for *V. cholerae* (28). These studies have shown that an extended exposure of stress led to changing requirements for successful resuscitation, indicating a second step of the VBNC state leading to a much deeper dormancy state, which might be applicable to our findings as well.

We found that the entry into the VBNC state was further triggered by desiccation, cold and hot temperatures, and anaerobiosis. The latter is of special interest, since *A. baumannii* has been found in the gastrointestinal tract as well as in anaerobic digesters (29, 30). Our findings may indicate that the strict aerobe survives anaerobiosis in a dormancy state and that the cells are not proliferating in the human gut or anaerobic digestors. This was not only the case for ATCC 19606^T^ but also found with more relevant strains including clinical isolates.

The identification of a 1-year-resuscitation window and the verification of the VBNC state in more virulent strains increase the threat *A. baumannii* imposes to humans. Although their metabolic activity decreases, many pathogens, such as *Campylobacter jejuni* and *Listeria monocytogenes*, were found either to be still virulent or to resuscitate under host conditions, initiating a relapse of infection (21, 31, 32). Our transcriptomic data revealed upregulation of four TetR-type transcriptional regulators that, when overexpressed, switch the culture from an opaque, virulent state to a translucent, avirulent state (33). Whether or not *A. baumannii* remains virulent in the VBNC state remains to be identified.

Genome-wide expression profiling gave further insights in the putative characteristics of VBNC *A. baumannii* cells. Several genes of the central carbon metabolism (TCA cycle) and of the respiratory chain (e.g., NADH dehydrogenase) are repressed, indicating a slowdown of metabolic activity as it is also described for VBNC *Vibrio* species (34, 35) and *Pseudomonas syringae* (36). In addition, we observed an upregulation of the genes associated with cell adhesion, like the trimeric autotransporter Ata. Some organisms, including VBNC *Shigella dysenterie* (37) and *Vibrio cholerae* (38), were described to maintain their ability to adhere to eukaryotic cells with a reduced scope which can also be assumed for *A. baumannii*.

The VBNC state is a novel, fundamental survival mechanism that allows *A. baumannii* to persist under unfavorable conditions for a long time. Unfortunately, the molecular basis of its induction is unknown. Our genome-wide expression profiling revealed various efflux pumps, DNA damage repair, and protection genes that were highly upregulated, but also HTZ92_1883 which is annotated as “stress-induced protein.” This gene was recently identified to be an “intrinsically disordered protein” (IDP) (19, 20) and characterized to be essential for desiccation tolerance in *A. baumannii* AB5075. Our transcriptomic data also revealed a downregulation of many proteases in VBNC cells of *A. baumannii*, including Lon and Clp. It is tempting to speculate that protein aggregates are accumulated in VBNC *A. baumannii* cells due to the loss of efficient protein degradation processes on one hand and the overproduction of IDRs on the other. This hypothesis is further supported by the finding that heat stress which is known to increase protein misfolding accelerates the VBNC-state entry in already stressed cells dramatically, while cold stress preserved culturability. Supported by the findings in *E. coli*, where different maturation levels of protein aggregates could be associated with the development of the VBNC state (39), the recognition of IDRs and regulatory protein aggregates may open a new perspective on stress resistance regulation in bacteria in general and maybe in VBNC-state induction in particular.

To date, detection of bacteria by plating is still regarded as the “gold standard” in the clinics or food industry (21). Due to the increasing number of bacteria known to go into the VBNC state which easily evade this classical detection method, new culture-independent methods for detection are urgently needed (21, 40). Although there are promising new techniques which use PCR in combination with viability markers, such as ethidium monoazide (EMA) and propidium monoazide (PMA) stains, to overcome the limitations of the standard PCR procedure, these methods are not yet used comprehensively (40, 41). In addition, generally applicable target sequences for identification of VBNC-state cells are lacking. The transcriptome analyses of VBNC *A. baumannii* ATCC 19606^T^ presented here may provide suitable candidates. One promising target may be iron siderophore biosynthesis, which not only is highly upregulated in *A. baumannii* but was also among the top-ranked DEGs observed in other bacteria in the VBNC state, such as *Pseudomonas syringae* and *V. cholerae* (34, 36).

## Materials and Methods

### Bacterial strains and culture conditions

*A. baumannii* ATCC 19606^T^, AB5075, ACICU, and AYE-T (42) as well as the clinical isolates CS36, CS121, and SC1946 were used; the clinical isolates were kindly provided by Dr. Stefan Göttig (University hospital, Frankfurt am Main, Germany). Depending on the experimental procedure, cells were grown either in mineral medium with 20 mM succinate as sole carbon and energy source (9) or in complex medium (LB) (43).

### Culturability assays and VBNC-state induction

The culturability and VBNC-state induction of *A. baumannii* strains were analyzed either under high-salt condition, desiccation, anaerobiosis, or temperature stress. High-salt conditions were imposed as described (11). The desiccation assay was performed as described (44). To study anaerobiosis, bacteria were grown overnight in LB medium, harvested, and adjusted to an OD_600nm_ = 4 with LB. Serum bottles containing 45 mL LB were purged with N_2_ for 20 min to remove oxygen prior to inoculation with 5 mL cell suspension. Serum bottles were then incubated at 37°C. To apply temperature stress, cells were pre-grown in mineral medium with 20 mM succinate as carbon source to the stationary growth phase. Cells were harvested and washed twice in sterile saline, and the suspension was adjusted to OD_600nm_ = 2 and a total volume of 50 mL. Cold and heat stresses were applied by incubation at 4°C and 42°C, respectively.

### Resuscitation assay

As soon as the CFU/mL of a cell suspension dropped below 100, the culture was addressed as non-culturable. At this point, the resuscitation assay was performed as described (11). Briefly, a sample of the unculturable cell suspension was diluted 1:10 in sterile phosphate-buffered saline (PBS, 140 mM NaCl, 10 mM KCl, 16 mM Na_2_HPO_4_, 2 mM KH_2_PO_4_) and incubated for 2 days on a rotary shaker at 37°C. Samples were plated on LB agar plates immediately after dilution in PBS and also after incubation for 2 days in PBS. Plates were incubated overnight and the colony-forming units were determined.

### Analysis of LIVE/DEAD staining by flow cytometry and fluorescence microscopy

The viability of a cell culture was determined using the LIVE/DEAD BacLight Bacterial Viability Kit (Thermo Fisher Scientific Inc., Hennigsdorf, Germany). Staining was performed according to the manufacturer’s instructions using the fluorescent stains Syto9 and propidium iodide. For quantification purposes, flow cytometry analyses were conducted using the Flow Cytometer BD FACSVerse (BD Biosciences, Franklin Lakes, NJ, USA). SYTO 9 and propidium iodide were both excited by a laser at 488 nm, and fluorescence was collected by a 527/32-nm (FITC-A cytogram) or 700/54-nm (PerCP-Cy5.5-A cytogram) bandpass filter, respectively. Populations were set (P5 = dead population, P6 = live population, P7 = damaged population, and P8 = unknown population) according to the populations received with the culturable- (late-exponentially grown) and dead-control (isopropanol-inactivated) cells. Viable cells (populations 6 and 7), and total cells (populations 5 to 8), were quantified using the kit’s microsphere standard. In addition to the quantitative flow cytometry analysis, stained cells were further qualitatively assessed by fluorescence microscopy (Zeiss Axio Imager M1) using a 63× objective.

### Transmission electron microscopy

All samples were centrifuged (10 min, 8,000 × *g*); each pellet was resuspended in 30 µL Ampuwa (Fresenius Kabi GmbH, Bad Homburg, Germany), negatively stained with 0.5% phosphotungstic acid (45), and finally imaged by TEM (Tecnai 12 Spirit, FEI, Eindhoven, Netherlands) at an acceleration voltage of 120 kV. Images have been adjusted for optimal brightness and contrast (applied to the entire image) using Adobe Photoshop (Adobe Systems, San Jose, CA, USA).

### CTC staining and confocal laser scanning microscopy

Cells were stained with the CTC Vitality Kit (BacLight RedoxSensor CTC Vitality Kit, Thermo Fisher Scientific Inc., Hennigsdorf, Germany) according to the manufacturer’s instructions. Actively respiring cells metabolize CTC to formazan which emits a purple fluorescence. Therefore, dead cells will appear green and viable cells purple/magenta. Fluorescence was visualized using confocal laser scanning microscopy (CLSM). After staining, samples were fixed in 2% fresh paraformaldehyde. Subsequently, all samples were counterstained with DAPI and mounted on glass slides and at least three representative spots with standardized areas were imaged with the CLSM (LSM780, Carl Zeiss AG, Oberkochen, Germany) using the Plan-Apochromat 63×/1.4 Oil DIC objective lens. All bacteria and bacteria with CTC staining were counted afterward using ImageJ (U.S. National Institutes of Health, Bethesda, MD, USA) and percentages calculated.

### Transcriptome analysis and statistics

RNA extraction and library preparation were performed as described (4). The Illumina Ribo-Zero plus rRNA Depletion Kit (Illumina, San Diego, CA, USA) was used in combination with a *A. baumanni* ATCC 19606^T^-specific probe to reduce the amount of rRNA-derived sequences. Sequencing of samples was performed on the NovaSeq 6000 instrument (Illumina) with the NovaSeq 6000 SP Reagent Kit v1.5 (100 cycles), and the NovaSeq XP 2-Lane Kit v1.5 was used in the paired-end mode and 2 × 61 cycles. For quality filtering and removing of the remaining adaptor sequences, trimmomatic-0.39 (46) and a cutoff phred-33 score of 15 were used. The mapping against the reference genomes of *A. baumannii* ATCC 19606^T^ (Ref: NZ_CP058289.1) was performed with Salmon (v1.9.0) (47). As mapping backbone, a file that contains all annotated transcripts excluding rRNA genes and the whole genome of the references as decoy was prepared with a k-mer size of 11. Decoy-aware mapping was done in the selective-alignment mode with “--mimicBT2,” “--disableChainingHeuristic,” and ”--recoverOrphans” flags as well as sequence and position bias correction and 10,000 bootstraps. For --fldMean and --fldSD, values of 325 and 25 were used, respectively. The quant.sf files produced by Salmon were subsequently loaded into R (v4.2.0) (R Core Team, 2020) using the tximport package (v1.24.0) (48). DeSeq2 (v1.36.0) (49) was used for normalization of the reads, and fold change shrinkages were also calculated with DeSeq2 (49) and the apeglm pack-age (v1.18.0) (50). Genes with a log_2_-fold change of ≥+2 and ≤−2 and a *P*_adjust_ < 0.05 were considered differentially expressed.

### Statistical analysis

Error bars denote the standard deviation of the mean derived from at least three independent biological replicates. Standard deviations were calculated and analyzed with Student’s *t*-test with GraphPad Prism Software. Data differences were considered significant if *P* ≤ 0.05.

## Data Availability

Gene expression data were deposited in the NCBI database (accession number: BioProject study SRP449893; samples SRR25305561-SRR25305578).

## SUPPLEMENTAL MATERIAL

Supplemental figures and tables mbio.02139-23-s0001.docx Tables S1 and S2 and Fig. S1 to S10.

ASM does not own the copyrights to Supplemental Material that may be linked to, or accessed through, an article. The authors have granted ASM a non-exclusive, world-wide license to publish the Supplemental Material files. Please contact the corresponding author directly for reuse.
