# Supplementary material for: The VBNC state: a fundamental survival strategy of Acinetobacter baumannii
Source: mBio. 2023 Sep 28;14(5):e02139-23. doi: 10.1128/mbio.02139-23 (PMC10653857; doi:10.1128/mbio.02139-23)
Supplement: Supplemental figures and tables — Tables S1 and S2 and Fig. S1 to S10. [file mbio.02139-23-s0001.docx]

**Supplemental Material**

**The VBNC state: a fundamental survival strategy of *Acinetobacter baumannii***

**Patricia König^a^, Alexander Wilhelm^b^, Christoph Schaudinn^c^, Anja Poehlein^d^, Rolf Daniel^d^, Marek Widera^b^, Beate Averhoff^a^, Volker Müller^a^***

*^a^Department of Molecular Microbiology & Bioenergetics, Institute of Molecular Biosciences, Goethe-University, Frankfurt am Main, Germany*

*^b^Institute for Medical Virology, University Hospital Frankfurt, Goethe University, Frankfurt am Main, Germany*

*^c^Advanced Light and Electron Microscopy ZBS4, Robert-Koch-Institute, Berlin, Germany*

*^d^Department of Genomic and Applied Microbiology & Göttingen Genomics Laboratory, Institute of Microbiology and Genetics, Georg-August University of Göttingen, Göttingen, Germany*

Running title: VBNC state in *A. baumannii*

Keywords: pathogen, virulence, persistence, desiccation

*Correspondence to: Volker Müller, Department of Molecular Microbiology & Bioenergetics, Institute of Molecular Biosciences, Goethe-University Frankfurt am Main, Max-von-Laue-Str. 9, 60438 Frankfurt, Germany. Tel.: + 49 69 79829507. E-mail address: [vmueller@bio.uni-frankfurt.de](mailto:a.corcelli@uniba.it)

**Tab. S1. Differentially regulated genes in VBNC cells.**

| **LocusTag** | **Annotation** | **log_2_ fold change** | **p-adjust value** |
| --- | --- | --- | --- |
| HTZ92_1018 | TetR family transcriptional regulator | 7,814490518 | 7,12795E-06 |
| HTZ92_2255 | antitermination protein | 7,761560641 | 6,22702E-06 |
| HTZ92_2850 | endoribonuclease L-PSP | 7,493878673 | 3,12329E-05 |
| HTZ92_2259 | hypothetical protein | 7,056434638 | 4,90084E-05 |
| HTZ92_3653 | hypothetical protein | 6,807946293 | 7,76E-16 |
| HTZ92_2309 | acyl-CoA dehydrogenase | 6,629028311 | 8,10516E-07 |
| HTZ92_2396 | hypothetical protein | 6,624600093 | 0,000315538 |
| HTZ92_3652 | DNA replication protein | 6,486558076 | 4,08E-23 |
| HTZ92_1240 | hypothetical protein | 6,387217814 | 1,97565E-06 |
| HTZ92_1518 | 2,3-dihydro-2,3-dihydroxybenzoate dehydrogenase | 6,37534614 | 2,45E-34 |
| HTZ92_3648 | hypothetical protein | 6,297123578 | 6,44508E-07 |
| HTZ92_0590 | YdjC-like protein | 6,294903339 | 2,13163E-06 |
| HTZ92_1466 | RND transporter | 6,149197402 | 1,02E-24 |
| HTZ92_0029 | hypothetical protein | 6,031751816 | 1,45226E-07 |
| HTZ92_2095 | hypothetical protein | 5,884634564 | 9,99304E-07 |
| HTZ92_3106 | DNA transfer protein p32 | 5,753494479 | 2,8304E-05 |
| HTZ92_2740 | transcriptional regulator, TetR family | 5,696785789 | 2,69673E-05 |
| HTZ92_1621 | hypothetical protein | 5,602341786 | 0,001843111 |
| HTZ92_1983 | aspartate:proton symporter | 5,54868381 | 2,08E-27 |
| HTZ92_3286 | hypothetical protein | 5,547472248 | 0,002430826 |
| rna-HTZ92_tRNA29 | NA | 5,460377478 | 0,002676936 |
| HTZ92_1623 | hypothetical protein | 5,422154755 | 0,002676936 |
| HTZ92_2592 | Hemerythrin | 5,409407007 | 6,29583E-05 |
| HTZ92_2747 | citrate:proton symporter | 5,409143118 | 2,33306E-08 |
| HTZ92_3634 | hypothetical protein | 5,339679012 | 4,44E-34 |
| HTZ92_2865 | TetR family transcriptional regulator | 5,326521539 | 9,859E-05 |
| HTZ92_2948 | TonB-dependent receptor | 5,315901236 | 2,87E-25 |
| HTZ92_1889 | hypothetical protein | 5,298757614 | 0,000192457 |
| HTZ92_1750 | FimA | 5,295383796 | 3,49E-15 |
| HTZ92_0872 | hypothetical protein | 5,221908491 | 2,06815E-08 |
| HTZ92_1016 | phosphohydrolase | 5,186519208 | 3,15804E-05 |
| HTZ92_0018 | tetratricopeptide repeat protein | 5,127031664 | 5,71E-19 |
| HTZ92_3225 | metal ABC transporter ATP-binding protein | 5,119069606 | 5,18E-23 |
| HTZ92_3647 | hypothetical protein | 5,090648174 | 9,56E-19 |
| HTZ92_0030 | hypothetical protein | 5,076541602 | 3,90167E-06 |
| HTZ92_1729 | GrpB protein | 4,974771175 | 3,4758E-05 |
| HTZ92_3640 | DNA replication protein | 4,959227928 | 1,20E-19 |
| HTZ92_1885 | NAD(P)-dependent oxidoreductase | 4,896726872 | 1,31E-18 |
| HTZ92_2570 | transcriptional regulator | 4,812660919 | 0,006378395 |
| HTZ92_2029 | transcriptional regulator | 4,808589811 | 9,94761E-06 |
| HTZ92_3370 | hypothetical protein | 4,704518118 | 0,007606264 |
| HTZ92_2554 | heat shock factor binding 1 family protein | 4,696043813 | 0,008680719 |
| HTZ92_1608 | hypothetical protein | 4,657791598 | 0,00976636 |
| HTZ92_1445 | GNAT family acetyltransferase | 4,610821221 | 2,06772E-06 |
| HTZ92_2312 | Thioesterase superfamily | 4,607061452 | 0,007186414 |
| HTZ92_3350 | phosphoglucosamine mutase | 4,60697572 | 1,17E-21 |
| HTZ92_3349 | Dihydropteroate synthase type-2 | 4,532492735 | 3,08E-24 |
| HTZ92_0893 | hypothetical protein | 4,528483675 | 2,61415E-07 |
| HTZ92_1961 | TetR family transcriptional regulator | 4,525268603 | 0,000211357 |
| HTZ92_1932 | monomeric sarcosine oxidase | 4,513561627 | 1,41143E-05 |
| HTZ92_1105 | gamma-glutamylputrescine synthetase | 4,495548989 | 4,3317E-08 |
| HTZ92_2372 | Inovirus Gp2 | 4,49000214 | 1,54773E-08 |
| HTZ92_3633 | Sel1 repeat protein | 4,486527002 | 1,37E-26 |
| HTZ92_1116 | hypothetical protein | 4,472791159 | 8,34424E-06 |
| HTZ92_1610 | hypothetical protein | 4,441101176 | 0,002363003 |
| HTZ92_2059 | hypothetical protein | 4,434480485 | 6,3964E-05 |
| HTZ92_0769 | nonribosomal peptide synthase | 4,355100197 | 5,40E-13 |
| HTZ92_1476 | transcriptional regulator, TetR family | 4,332364059 | 0,00010341 |
| HTZ92_0779 | isochorismatase | 4,306286144 | 2,2771E-06 |
| HTZ92_3638 | plasmid mobilization protein | 4,290111173 | 1,29E-12 |
| HTZ92_2784 | p-aminobenzoate synthetase | 4,272536147 | 1,89543E-07 |
| HTZ92_3343 | hypothetical protein | 4,247295712 | 7,77E-12 |
| HTZ92_3284 | 3-oxoacyl-ACP synthase | 4,237781616 | 1,40E-17 |
| HTZ92_2273 | hypothetical protein | 4,210971272 | 0,003095406 |
| HTZ92_0562 | phage-related reverse | 4,205641865 | 3,13945E-08 |
| HTZ92_2930 | hypothetical protein | 4,20196955 | 0,014522397 |
| HTZ92_1477 | hypothetical protein | 4,170468523 | 0,003461668 |
| HTZ92_0773 | iron ABC transporter ATP-binding protein | 4,151916984 | 1,02811E-06 |
| HTZ92_3098 | selenocysteine synthase | 4,148967421 | 1,0909E-05 |
| HTZ92_0809 | hypothetical protein | 4,122106071 | 0,016569312 |
| HTZ92_0413 | bacteriolytic lipoprotein entericidin B | 4,118546637 | 3,71804E-05 |
| HTZ92_2870 | hypothetical protein | 4,107504764 | 9,42383E-06 |
| HTZ92_3493 | MATE efflux family protein | 4,08051787 | 0,00117084 |
| HTZ92_2377 | hypothetical protein | 4,078101854 | 1,56243E-09 |
| HTZ92_0890 | glutamate 5-kinase | 4,075505638 | 2,85583E-08 |
| HTZ92_2158 | amino acid transporter LysE | 4,018075833 | 0,000337189 |
| HTZ92_0767 | acinetobactin utilization protein | 4,012016486 | 1,84E-11 |
| HTZ92_0405 | Gram-positive signal peptide protein, YSIRK family | 4,003970417 | 1,07604E-05 |
| HTZ92_2293 | transferase | 3,985115248 | 3,7155E-05 |
| HTZ92_1456 | hypothetical protein | 3,964215471 | 1,41433E-05 |
| HTZ92_1413 | TetR family transcriptional regulator | 3,955396373 | 0,000138468 |
| HTZ92_0904 | antirepressor | 3,927800862 | 2,523E-06 |
| HTZ92_2732 | TetR family transcriptional regulator | 3,914923109 | 3,25806E-10 |
| HTZ92_2081 | hypothetical protein | 3,910060217 | 4,41245E-09 |
| HTZ92_3341 | hypothetical protein | 3,894516326 | 1,03E-18 |
| HTZ92_0109 | type IV fimbrial biogenesis protein PilV | 3,861937401 | 9,23781E-06 |
| HTZ92_3226 | DNA repair protein | 3,834887969 | 1,6643E-09 |
| HTZ92_1696 | type 4 fimbrial biogenesis protein | 3,830448823 | 0,000302119 |
| HTZ92_1470 | RND transporter | 3,83024553 | 3,02986E-08 |
| HTZ92_2413 | 5-formyltetrahydrofolate cyclo-ligase | 3,828450325 | 0,000100323 |
| HTZ92_0113 | pilin | 3,825049982 | 0,000106103 |
| HTZ92_3087 | transposase | 3,82357122 | 0,001305389 |
| HTZ92_0587 | dolichyl-phosphate-mannose--protein mannosyltransferase | 3,809464076 | 1,66711E-06 |
| HTZ92_3190 | hypothetical protein | 3,800775389 | 6,42414E-09 |
| HTZ92_2559 | hypothetical protein | 3,788745395 | 2,36828E-06 |
| HTZ92_2276 | hypothetical protein | 3,785741344 | 0,00094393 |
| HTZ92_3254 | acyl-CoA synthetase | 3,780663294 | 2,08E-17 |
| HTZ92_3466 | hypothetical protein | 3,77408887 | 0,00044312 |
| HTZ92_1884 | hypothetical protein | 3,770655193 | 0,004947057 |
| HTZ92_2398 | hemin receptor | 3,753081884 | 0,001981105 |
| HTZ92_2249 | hypothetical protein | 3,74697142 | 4,70452E-05 |
| HTZ92_2567 | hypothetical protein | 3,737770406 | 0,00026553 |
| HTZ92_1278 | hypothetical protein | 3,732663838 | 2,4787E-09 |
| HTZ92_1030 | dehydrogenase | 3,731715221 | 5,18656E-06 |
| HTZ92_2138 | hypothetical protein | 3,696735126 | 5,2462E-05 |
| HTZ92_0917 | hypothetical protein | 3,624287812 | 0,000399203 |
| HTZ92_1131 | hypothetical protein | 3,620195483 | 0,022687713 |
| HTZ92_2264 | hypothetical protein | 3,60441501 | 3,46465E-05 |
| HTZ92_3635 | organic hydroperoxide resistance protein | 3,589206172 | 2,83563E-09 |
| HTZ92_1453 | hypothetical protein | 3,58085511 | 0,003920322 |
| HTZ92_0900 | hypothetical protein | 3,550794754 | 1,38057E-08 |
| HTZ92_0674 | exconuclease ABC subunit B | 3,547708813 | 1,39E-19 |
| HTZ92_2541 | PAAR motif | 3,539716194 | 1,40E-11 |
| HTZ92_1486 | dienelactone hydrolase family protein | 3,527337511 | 6,15409E-07 |
| HTZ92_0328 | TonB-dependent receptor | 3,523744683 | 1,30221E-10 |
| HTZ92_0998 | threonine transporter | 3,518670211 | 0,000581649 |
| HTZ92_0471 | SAM-dependent methyltransferase | 3,50496548 | 0,000774877 |
| HTZ92_0897 | hypothetical protein | 3,49492038 | 2,6679E-06 |
| HTZ92_2232 | hypothetical protein | 3,492418373 | 0,000150976 |
| HTZ92_2745 | lysine transporter LysE | 3,487757049 | 0,00184026 |
| HTZ92_0028 | restriction endonuclease PvuRts1I | 3,454905727 | 1,61046E-07 |
| HTZ92_2239 | signal peptide protein | 3,442906778 | 4,03454E-09 |
| HTZ92_0704 | MFS transporter | 3,44024193 | 3,88481E-05 |
| HTZ92_1531 | carboxymethylenebutenolidase | 3,432428196 | 2,09667E-08 |
| HTZ92_3206 | DNA protecting protein DprA | 3,427572174 | 1,57103E-06 |
| HTZ92_2455 | sporulation and cell division repeat protein | 3,426671992 | 3,58156E-06 |
| HTZ92_0745 | hypothetical protein | 3,421329461 | 0,004256636 |
| HTZ92_1775 | acyltransferase | 3,41008919 | 6,01879E-09 |
| HTZ92_1868 | lysine transporter LysE | 3,407589075 | 0,00131402 |
| HTZ92_2310 | porin | 3,407260456 | 2,91269E-06 |
| HTZ92_0237 | nitroreductase family protein | 3,386380399 | 0,001160187 |
| HTZ92_2375 | Pyocin activator protein PrtN | 3,385560309 | 0,006654638 |
| HTZ92_1159 | TetR family transcriptional regulator | 3,379315232 | 0,000189099 |
| HTZ92_0019 | hypothetical protein | 3,378142023 | 3,04037E-07 |
| HTZ92_0914 | hypothetical protein | 3,377974675 | 8,22089E-06 |
| HTZ92_1471 | hypothetical protein | 3,347298793 | 6,13952E-07 |
| HTZ92_1993 | dihydrodipicolinate synthetase | 3,346665641 | 4,77E-13 |
| HTZ92_0778 | 2,3-dihydroxybenzoate-AMP ligase | 3,33937643 | 6,77304E-10 |
| HTZ92_1557 | nitrate ABC transporter substrate-binding protein | 3,339337549 | 3,0814E-06 |
| HTZ92_0333 | glutathione S-transferase | 3,312319275 | 9,33139E-05 |
| HTZ92_2981 | hypothetical protein | 3,305332926 | 5,1513E-05 |
| HTZ92_2510 | aerobic C4-dicarboxylate transporter | 3,300931665 | 3,84528E-05 |
| HTZ92_2042 | hemagglutinin | 3,29883227 | 3,22129E-07 |
| HTZ92_0909 | hypothetical protein | 3,288741611 | 5,95766E-05 |
| HTZ92_3368 | hypothetical protein | 3,284048046 | 2,47614E-09 |
| HTZ92_2557 | hypothetical protein | 3,277315066 | 1,1807E-05 |
| HTZ92_1604 | secretion protein HlyD | 3,263438847 | 9,25843E-10 |
| HTZ92_2257 | hypothetical protein | 3,261034691 | 3,88481E-05 |
| HTZ92_3654 | hypothetical protein | 3,258075981 | 5,37E-13 |
| HTZ92_3636 | MarR family transcriptional regulator | 3,237330487 | 1,12583E-08 |
| HTZ92_1749 | hypothetical protein | 3,237176648 | 0,000989383 |
| HTZ92_2274 | hypothetical protein | 3,236382955 | 0,000280589 |
| HTZ92_2064 | transporter | 3,228027502 | 0,000858793 |
| HTZ92_0154 | LysR family transcriptional regulator | 3,227066288 | 1,63254E-06 |
| HTZ92_0982 | hypothetical protein | 3,225170451 | 8,18567E-05 |
| HTZ92_1464 | hypothetical protein | 3,216480629 | 3,7045E-06 |
| HTZ92_1949 | peptide synthetase | 3,202255904 | 3,04037E-07 |
| HTZ92_1044 | transporter, major facilitator family protein | 3,200452779 | 1,97565E-06 |
| HTZ92_2941 | hypothetical protein | 3,184404398 | 0,00732789 |
| HTZ92_2241 | hypothetical protein | 3,181517989 | 1,63883E-07 |
| HTZ92_1859 | LysR substrate binding domain-containing protein | 3,171167476 | 7,30801E-08 |
| HTZ92_3289 | hypothetical protein | 3,170168872 | 9,79438E-07 |
| HTZ92_1235 | hypothetical protein | 3,168981373 | 3,40534E-05 |
| HTZ92_1881 | DNA polymerase V | 3,155735973 | 1,07369E-07 |
| HTZ92_2935 | hypothetical protein | 3,139530468 | 4,22032E-06 |
| HTZ92_0535 | alkaline phosphatase | 3,138058707 | 2,6634E-07 |
| HTZ92_2304 | MarR family transcriptional regulator | 3,134125364 | 0,000853428 |
| HTZ92_3174 | hypothetical protein | 3,127702002 | 8,2837E-08 |
| HTZ92_3283 | signal peptide protein | 3,126330716 | 6,23057E-08 |
| HTZ92_2311 | hypothetical protein | 3,126064429 | 0,001583321 |
| HTZ92_1493 | thiamine ABC transporter permease | 3,116416219 | 2,14481E-10 |
| HTZ92_2135 | hypothetical protein | 3,111025844 | 0,001517768 |
| HTZ92_1203 | TetR family transcriptional regulator | 3,110167948 | 0,000270748 |
| HTZ92_2540 | zinc protease | 3,10685055 | 1,26374E-06 |
| HTZ92_1778 | porin | 3,097373568 | 2,34683E-08 |
| HTZ92_0303 | DNA repair protein | 3,095923874 | 0,002081452 |
| HTZ92_0889 | hypothetical protein | 3,087131098 | 0,000124225 |
| HTZ92_0784 | thioesterase | 3,078842959 | 3,87699E-05 |
| HTZ92_2415 | Coiled stalk of trimeric autotransporter adhesin | 3,072521923 | 6,34371E-10 |
| HTZ92_0025 | hypothetical protein | 3,071234698 | 5,11009E-06 |
| HTZ92_1620 | siderophore biosynthesis protein | 3,064643853 | 5,95156E-07 |
| HTZ92_2326 | hypothetical protein | 3,059384786 | 0,001146098 |
| HTZ92_1167 | porin | 3,041089739 | 1,33E-12 |
| HTZ92_3639 | DNA replication protein | 3,034515123 | 7,78707E-09 |
| HTZ92_0874 | hypothetical protein | 3,027821056 | 0,001001407 |
| HTZ92_1330 | metal-dependent hydrolase | 3,025115234 | 6,69303E-05 |
| HTZ92_3255 | LuxR family transcriptional regulator | 3,01987896 | 9,51415E-06 |
| HTZ92_1480 | hypothetical protein | 3,01230849 | 0,00043786 |
| HTZ92_1160 | TonB dependent receptor | 3,008043616 | 8,96152E-09 |
| HTZ92_3253 | acyl-CoA dehydrogenase | 3,007477548 | 2,36585E-07 |
| HTZ92_2560 | hypothetical protein | 2,982544207 | 1,32189E-06 |
| HTZ92_2751 | tricarballylate utilization protein B | 2,981130523 | 0,000167034 |
| HTZ92_1883 | stress-induced protein | 2,979671013 | 3,17298E-09 |
| HTZ92_1668 | ABC transporter, substrate-binding protein, family 5 | 2,962088638 | 1,42364E-09 |
| HTZ92_1570 | Fis family transcriptional regulator | 2,949728537 | 2,84571E-07 |
| HTZ92_1823 | hydrolase | 2,936715154 | 1,77162E-09 |
| HTZ92_0026 | HxlR family transcriptional regulator | 2,9294827 | 4,61046E-07 |
| HTZ92_3492 | MATE family efflux transporter | 2,928873891 | 9,54926E-05 |
| HTZ92_1752 | fimbrial usher protein | 2,927942607 | 3,72493E-07 |
| HTZ92_3632 | Cro/Cl family transcriptional regulator | 2,91678607 | 2,69241E-10 |
| HTZ92_2376 | transcriptional regulator | 2,915853785 | 0,002053774 |
| HTZ92_2236 | hypothetical protein | 2,912202414 | 3,58156E-06 |
| HTZ92_2833 | transporter | 2,902482418 | 0,00426281 |
| HTZ92_0533 | hypothetical protein | 2,898464341 | 0,007147306 |
| HTZ92_2324 | hypothetical protein | 2,892967421 | 0,015239553 |
| HTZ92_0263 | TetR family transcriptional regulator | 2,891101303 | 4,18348E-05 |
| HTZ92_1282 | alpha/beta hydrolase | 2,888410101 | 1,66964E-07 |
| HTZ92_2237 | hypothetical protein | 2,874324791 | 0,009940865 |
| HTZ92_2004 | ankyrin repeat protein | 2,855039135 | 0,001210377 |
| HTZ92_3003 | LysR family transcriptional regulator | 2,838714815 | 6,36251E-06 |
| HTZ92_3257 | N-acylhomoserine lactone synthase | 2,833549528 | 3,01779E-05 |
| HTZ92_2242 | phage-like protein | 2,822189538 | 4,37193E-08 |
| HTZ92_3147 | tyrosine recombinase XerD | 2,82103319 | 1,92982E-06 |
| HTZ92_0702 | hypothetical protein | 2,812925416 | 0,004659257 |
| HTZ92_0098 | fimbrial protein | 2,812522791 | 0,011961759 |
| HTZ92_2223 | hypothetical protein | 2,804130356 | 4,46349E-05 |
| HTZ92_1212 | histidine kinase | 2,798692303 | 0,001787419 |
| HTZ92_0379 | type 4 fimbrial biogenesis protein FimT | 2,789256066 | 0,000302119 |
| HTZ92_0027 | NAD(P)H oxidoreductase | 2,785672931 | 8,68823E-06 |
| HTZ92_0550 | glycerophosphodiester phosphodiesterase family protein | 2,782094507 | 8,73108E-07 |
| HTZ92_3011 | 50S ribosomal protein L31 | 2,781551214 | 8,65375E-07 |
| HTZ92_2974 | hypothetical protein | 2,777956306 | 5,63867E-07 |
| HTZ92_3601 | isochorismatase | 2,774007353 | 0,001457801 |
| HTZ92_2033 | flavodoxin | 2,756389375 | 0,000886183 |
| HTZ92_3367 | iron-regulated element | 2,75228233 | 0,00065373 |
| HTZ92_0777 | acinetobactin biosynthesis protein | 2,740173624 | 1,20136E-06 |
| HTZ92_2748 | substrate-binding protein | 2,733639796 | 0,00066099 |
| HTZ92_0879 | phage protein NinB | 2,729897332 | 0,000121058 |
| HTZ92_3409 | peptidase | 2,724654436 | 0,000185867 |
| HTZ92_0760 | molybdenum cofactor biosynthesis protein MoeA | 2,719412069 | 9,33139E-05 |
| HTZ92_3342 | hypothetical protein | 2,718997588 | 0,027919301 |
| HTZ92_2233 | hypothetical protein | 2,716397743 | 4,69317E-06 |
| HTZ92_3502 | CobW/P47K family protein | 2,711313093 | 4,12042E-08 |
| HTZ92_0883 | hypothetical protein | 2,70728779 | 2,36523E-08 |
| HTZ92_3617 | hypothetical protein | 2,706900392 | 0,006541861 |
| rna-HTZ92_tRNA71 | NA | 2,702631059 | 0,035421251 |
| HTZ92_2176 | benzoate 1,2-dioxygenase subunit beta | 2,702270723 | 0,001910766 |
| HTZ92_1789 | methionine ABC transporter ATP-binding protein | 2,698659692 | 0,000148835 |
| HTZ92_0941 | hemin transporter HemP | 2,696528002 | 0,000975206 |
| HTZ92_2234 | hypothetical protein | 2,69514424 | 1,96397E-07 |
| HTZ92_1494 | hypothetical protein | 2,691683157 | 0,010791025 |
| HTZ92_0898 | tail fiber protein | 2,691342741 | 4,35044E-08 |
| HTZ92_2139 | hypothetical protein | 2,687668306 | 0,001419641 |
| HTZ92_1984 | NADP-dependent fatty aldehyde dehydrogenase | 2,685673062 | 1,89543E-07 |
| HTZ92_2193 | hemolysin-type calcium-binding repeat (2 copies) | 2,684261369 | 6,93204E-07 |
| HTZ92_3354 | Arsenic resistance protein ArsH | 2,681512579 | 3,04037E-07 |
| HTZ92_1927 | cag pathogenicity island protein Cag19 | 2,67539846 | 1,96E-11 |
| HTZ92_2134 | DNA-binding protein | 2,673748679 | 0,014362713 |
| HTZ92_0224 | acyl-CoA dehydrogenase | 2,671427613 | 3,08489E-06 |
| HTZ92_3327 | phospholipase C precursor (PLC) (Phosphatidylcholine cholinephosphohydrolase) | 2,664702424 | 3,28796E-09 |
| HTZ92_2750 | tricarballylate dehydrogenase | 2,662681796 | 7,48799E-05 |
| HTZ92_2203 | hypothetical protein | 2,660639105 | 6,63031E-05 |
| HTZ92_2222 | glycosyl hydrolase | 2,66016233 | 7,67125E-05 |
| HTZ92_3195 | FMN reductase | 2,652642715 | 0,001102247 |
| HTZ92_2552 | hypothetical protein | 2,650022133 | 0,004507986 |
| HTZ92_2221 | anaerobic dehydrogenase | 2,649691539 | 0,003296433 |
| HTZ92_1484 | short-chain dehydrogenase | 2,647027883 | 2,34853E-06 |
| HTZ92_0882 | terminase | 2,640660702 | 8,76071E-07 |
| HTZ92_3556 | OHCU decarboxylase | 2,640274747 | 0,003117492 |
| HTZ92_0737 | hypothetical protein | 2,63637613 | 0,004956759 |
| HTZ92_2985 | 3-isopropylmalate dehydratase | 2,634497948 | 1,11E-11 |
| HTZ92_3078 | hypothetical protein | 2,633656577 | 0,032846589 |
| HTZ92_1751 | pilus assembly protein | 2,633194798 | 0,000337189 |
| HTZ92_3105 | DNA transfer protein p32 | 2,626239572 | 2,41228E-05 |
| HTZ92_3511 | X-Pro dipeptidyl-peptidase | 2,613567942 | 2,06815E-08 |
| HTZ92_2153 | hypothetical protein | 2,613451694 | 0,000122285 |
| HTZ92_1140 | transcriptional regulator | 2,609899395 | 1,38446E-07 |
| HTZ92_0923 | MexE family multidrug efflux RND transporter periplasmic adaptor subunit | 2,60957751 | 0,00066099 |
| HTZ92_0771 | iron ABC transporter permease | 2,60797172 | 6,29583E-05 |
| HTZ92_0675 | outer membrane lipoprotein Blc | 2,602915782 | 8,97E-11 |
| HTZ92_1442 | iron transporter | 2,600950141 | 0,000415049 |
| HTZ92_1468 | hypothetical protein | 2,591754645 | 7,45593E-06 |
| HTZ92_0766 | DNA-binding protein | 2,590217783 | 1,63832E-06 |
| HTZ92_0086 | Pilus assembly protein, PilO | 2,574780173 | 0,00105359 |
| HTZ92_1640 | transcriptional regulator HU subunit α | 2,569778796 | 2,06021E-05 |
| HTZ92_0022 | TetR family transcriptional regulator | 2,556636406 | 0,00354003 |
| HTZ92_3559 | ankyrin repeat protein | 2,554309361 | 2,54842E-06 |
| HTZ92_1421 | C4-dicarboxylate ABC transporter | 2,549561326 | 6,33891E-05 |
| HTZ92_2141 | hypothetical protein | 2,547568807 | 0,006793361 |
| HTZ92_2300 | MFS transporter | 2,547374327 | 2,99278E-07 |
| HTZ92_2815 | hypothetical protein | 2,545376369 | 4,03537E-06 |
| HTZ92_0156 | HrgA protein | 2,544696789 | 0,000237181 |
| HTZ92_2143 | hypothetical protein | 2,539302912 | 2,35543E-06 |
| HTZ92_1523 | anthranilate 1,2-dioxygenase large subunit | 2,527330738 | 1,49078E-05 |
| HTZ92_1479 | acetyltransferase | 2,527027162 | 0,000496261 |
| HTZ92_2564 | hypothetical protein | 2,526572522 | 0,011161298 |
| HTZ92_2539 | hypothetical protein | 2,516516955 | 8,45598E-05 |
| HTZ92_1609 | peptidase | 2,505556799 | 3,81292E-05 |
| HTZ92_1849 | triphosphoribosyl-dephospho-CoA synthase MdcB | 2,502935017 | 2,58368E-06 |
| HTZ92_3631 | hypothetical protein | 2,502888975 | 2,98478E-10 |
| HTZ92_0772 | iron ABC transporter permease | 2,497974595 | 0,00057748 |
| HTZ92_1029 | stearoyl-CoA 9-desaturase | 2,497596165 | 0,000928655 |
| HTZ92_1469 | GH3 auxin-responsive promoter | 2,495938317 | 2,03204E-06 |
| HTZ92_0985 | protein kinase | 2,493638463 | 0,002210918 |
| HTZ92_0763 | transcriptional regulator, TetR family | 2,493033369 | 0,004945692 |
| HTZ92_1590 | methyltransferase | 2,489036145 | 3,81292E-05 |
| HTZ92_2937 | zonular occludens toxin | 2,488820554 | 0,000674168 |
| HTZ92_3256 | hypothetical protein | 2,488093363 | 0,001178118 |
| HTZ92_1625 | demethylmenaquinone methyltransferase | 2,487866639 | 5,09131E-05 |
| HTZ92_0356 | protein-tyrosine-phosphatase | 2,487753666 | 0,003528307 |
| HTZ92_2845 | dehydrogenase | 2,487375253 | 0,000140098 |
| HTZ92_1266 | AsnC family transcriptional regulator | 2,486319891 | 0,010967121 |
| HTZ92_2344 | TetR family transcriptional regulator | 2,471933044 | 0,004250212 |
| HTZ92_1540 | hypothetical protein | 2,464881159 | 0,001233893 |
| HTZ92_3145 | ferrous iron transporter B | 2,464614168 | 2,29639E-08 |
| HTZ92_2248 | helicase | 2,458733622 | 2,36585E-07 |
| HTZ92_2260 | hypothetical protein | 2,456205919 | 7,70218E-05 |
| HTZ92_2366 | phage/plasmid-like protein TIGR03299 | 2,447392153 | 0,000124479 |
| HTZ92_3359 | hypothetical protein | 2,443241636 | 3,55579E-05 |
| HTZ92_0716 | RNA pseudouridine synthase | 2,441259271 | 6,22986E-07 |
| HTZ92_2224 | Putative phage tail protein | 2,436866787 | 1,16794E-07 |
| HTZ92_1895 | hypothetical protein | 2,43558996 | 0,020946995 |
| HTZ92_1400 | pyridine nucleotide-disulfide oxidoreductase | 2,433043846 | 1,0765E-07 |
| HTZ92_1526 | AraC family transcriptional regulator | 2,429815606 | 2,25202E-05 |
| HTZ92_0226 | hypothetical protein | 2,427866029 | 0,029357409 |
| HTZ92_2632 | phosphate porin | 2,419520763 | 0,001786726 |
| HTZ92_0332 | protein PsiE | 2,419121988 | 0,006839264 |
| HTZ92_2443 | hypothetical protein | 2,417499148 | 1,57068E-06 |
| HTZ92_1826 | NCS1 family APC transporter | 2,41722635 | 0,000571844 |
| HTZ92_2420 | NADP-specific glutamate dehydrogenase | 2,409680861 | 2,39873E-07 |
| HTZ92_1362 | MFS transporter | 2,401266051 | 4,46349E-05 |
| HTZ92_2587 | twitching motility protein PilT | 2,391902802 | 0,000159949 |
| HTZ92_3390 | ABC transporter permease | 2,389571274 | 0,00136131 |
| HTZ92_0016 | LysR family transcriptional regulator | 2,387565564 | 4,69317E-06 |
| HTZ92_2469 | sulfatase-modifying protein | 2,386888844 | 5,01588E-05 |
| HTZ92_1509 | renal dipeptidase family protein | 2,386665071 | 6,63031E-05 |
| HTZ92_2256 | hypothetical protein | 2,385056292 | 0,01734814 |
| HTZ92_1202 | PepSY-associated TM region | 2,366026235 | 2,8304E-05 |
| HTZ92_0875 | hypothetical protein | 2,365918592 | 0,002387149 |
| HTZ92_2755 | MFS transporter | 2,362274799 | 0,000197317 |
| HTZ92_2109 | hypothetical protein | 2,361537877 | 0,000342915 |
| HTZ92_1671 | TonB-dependent receptor | 2,359627075 | 3,53243E-05 |
| HTZ92_1636 | acyl-CoA dehydrogenase | 2,356584194 | 4,32213E-07 |
| HTZ92_1098 | 2-amino-4-hydroxy-6-hydroxymethyldihydropteridin e pyrophosphokinase | 2,353785362 | 0,001723408 |
| HTZ92_1865 | serine acetyltransferase | 2,351001053 | 5,84111E-05 |
| HTZ92_1631 | siderophore biosynthesis protein | 2,337992642 | 7,2778E-07 |
| HTZ92_0120 | lipase chaperone | 2,336012102 | 2,25202E-05 |
| HTZ92_2194 | hypothetical protein | 2,327431999 | 0,000473172 |
| HTZ92_2028 | Asp-tRNAAsn/Glu-tRNAGln amidotransferase A subunit | 2,327335305 | 2,46748E-06 |
| HTZ92_1164 | hypothetical protein | 2,3217334 | 0,034824855 |
| HTZ92_1515 | bifunctional metallophosphatase/5'-nucleotidase | 2,320486878 | 2,06772E-06 |
| HTZ92_0537 | methyltransferase | 2,319909591 | 0,005811222 |
| HTZ92_1855 | negative transcriptional regulator | 2,318500202 | 0,002584055 |
| HTZ92_1036 | DNA-binding response regulator | 2,318436559 | 0,00105359 |
| HTZ92_2534 | porin | 2,31407742 | 0,000203769 |
| HTZ92_0785 | 4'-phosphopantetheinyl transferase | 2,31256536 | 0,003415547 |
| HTZ92_0715 | isochorismatase | 2,312053057 | 2,59223E-05 |
| HTZ92_1489 | two-component system sensor histidine kinase | 2,31088156 | 9,16316E-05 |
| HTZ92_0260 | S-(hydroxymethyl)glutathione synthase | 2,309566183 | 0,00744382 |
| HTZ92_1819 | ArsR family transcriptional regulator | 2,309080706 | 0,012541975 |
| HTZ92_3513 | glutamate racemase | 2,307943787 | 6,09756E-06 |
| HTZ92_1522 | LysR family transcriptional regulator | 2,304543662 | 0,000425387 |
| HTZ92_2988 | hypothetical protein | 2,302948692 | 0,027474587 |
| HTZ92_1162 | hypothetical protein | 2,300009554 | 0,000497189 |
| rna-HTZ92_ffs | NA | 2,299460264 | 0,00043786 |
| HTZ92_1367 | polyketide cyclase | 2,299418412 | 0,007317282 |
| HTZ92_1944 | allantoin permease | 2,297442646 | 0,001278292 |
| HTZ92_1517 | 2,3-dihydro-2,3-dihydroxybenzoate synthetase | 2,293360958 | 2,91408E-05 |
| HTZ92_0162 | HPP family | 2,292942 | 4,64853E-06 |
| HTZ92_2584 | ATP-dependent dsDNA exonuclease | 2,29069473 | 2,87581E-06 |
| HTZ92_1085 | LuxR family transcriptional regulator | 2,290064883 | 0,000109372 |
| HTZ92_2849 | transporter | 2,289496561 | 0,005342806 |
| HTZ92_0181 | DNA transformation protein | 2,2882329 | 0,004353346 |
| HTZ92_2043 | hemolysin activator protein | 2,285915052 | 1,44523E-05 |
| HTZ92_1948 | alpha/beta hydrolase | 2,268678679 | 0,000127375 |
| HTZ92_2543 | hypothetical protein | 2,263311734 | 0,007830879 |
| HTZ92_3173 | dihydrodipicolinate synthase | 2,257202309 | 0,000425376 |
| HTZ92_1467 | MFS transporter | 2,256906458 | 0,000361476 |
| HTZ92_2065 | AsnC family transcriptional regulator | 2,253923728 | 0,013535438 |
| HTZ92_2099 | hypothetical protein | 2,253290071 | 1,1273E-07 |
| HTZ92_2975 | hypothetical protein | 2,251236756 | 5,70516E-05 |
| HTZ92_1364 | Glu-tRNA amidotransferase | 2,244426606 | 3,01779E-05 |
| HTZ92_0564 | TetR family transcriptional regulator | 2,243803362 | 0,008197532 |
| HTZ92_1825 | GntR family transcriptional regulator | 2,235532674 | 6,69968E-07 |
| HTZ92_1482 | hypothetical protein | 2,23551141 | 0,001312313 |
| HTZ92_0108 | fimbrial protein | 2,229555314 | 0,006959334 |
| HTZ92_2053 | haloacid dehalogenase | 2,229211369 | 0,005470662 |
| HTZ92_0761 | hypothetical protein | 2,225111129 | 0,008638373 |
| HTZ92_0191 | globin | 2,224030238 | 2,92049E-07 |
| HTZ92_3247 | phosphopantetheine-protein transferase | 2,223660201 | 0,003570353 |
| HTZ92_1478 | type III restriction endonuclease subunit R | 2,216582874 | 5,83732E-07 |
| HTZ92_2244 | hypothetical protein | 2,210541328 | 0,012135455 |
| HTZ92_1258 | recombinase RecX | 2,210210624 | 0,002194017 |
| HTZ92_0871 | hypothetical protein | 2,209889477 | 0,008462233 |
| HTZ92_2227 | hypothetical protein | 2,203346109 | 1,56647E-06 |
| HTZ92_0581 | alpha/beta hydrolase | 2,19752772 | 0,00481467 |
| HTZ92_2931 | phage replication initiation factor | 2,197176804 | 0,00066099 |
| HTZ92_0926 | histidine/lysine/arginine/ornithine ABC transporter permease HisQ | 2,190565826 | 0,007919161 |
| HTZ92_1854 | amino acid acetyltransferase | 2,188827235 | 0,000997469 |
| HTZ92_3240 | DoxX | 2,188249004 | 3,65212E-05 |
| HTZ92_1913 | porin | 2,182589735 | 8,08954E-05 |
| HTZ92_2061 | TetR family transcriptional regulator | 2,179396364 | 0,010173853 |
| HTZ92_0783 | ABC transporter | 2,161551021 | 8,62879E-05 |
| HTZ92_0774 | ferric anguibactin-binding protein | 2,160845854 | 0,00053183 |
| HTZ92_2151 | head morphogenesis protein | 2,158946079 | 0,000477552 |
| HTZ92_1630 | lysine/ornithine N-monooxygenase | 2,154938781 | 0,00023361 |
| HTZ92_0780 | histidine decarboxylase | 2,148439397 | 0,000223902 |
| HTZ92_0376 | quaternary ammonium transporter | 2,145557974 | 0,02182979 |
| HTZ92_2586 | Tfp pilus assembly protein, pilus retraction ATPase PilT | 2,143359538 | 3,01779E-05 |
| HTZ92_1928 | acyl-CoA dehydrogenase | 2,139019794 | 5,05865E-05 |
| HTZ92_3512 | haloacid dehalogenase | 2,137789202 | 0,00043702 |
| HTZ92_3172 | class II aldolase | 2,133654166 | 0,008287072 |
| HTZ92_3334 | hypothetical protein | 2,130877931 | 0,000774569 |
| HTZ92_3650 | hypothetical protein | 2,123549684 | 4,29024E-07 |
| HTZ92_0492 | FMN reductase | 2,117346458 | 6,21082E-05 |
| HTZ92_1748 | hypothetical protein | 2,112695264 | 0,017354107 |
| HTZ92_2238 | hypothetical protein | 2,102656621 | 5,59695E-05 |
| HTZ92_0470 | molecular chaperone DnaJ | 2,101638993 | 0,000446125 |
| HTZ92_1426 | Putative inner membrane exporter, YdcZ | 2,096101943 | 0,00658419 |
| HTZ92_0835 | branched-chain amino acid transporter | 2,079928611 | 0,044338321 |
| HTZ92_1918 | enoyl-CoA hydratase | 2,066467379 | 0,000127375 |
| HTZ92_1538 | DoxX | 2,06376651 | 0,014531084 |
| HTZ92_1556 | ABC transporter permease | 2,062312238 | 0,00415975 |
| HTZ92_2263 | hypothetical protein | 2,059634512 | 0,042057303 |
| HTZ92_0915 | lysozyme | 2,055943391 | 0,001714372 |
| HTZ92_2104 | hypothetical protein | 2,055102216 | 3,68965E-06 |
| HTZ92_3442 | RND transporter | 2,054297233 | 0,000116561 |
| HTZ92_0910 | bacteriophage protein | 2,040891484 | 0,001861712 |
| HTZ92_0884 | phage head morphogenesis protein | 2,040726056 | 7,64115E-05 |
| HTZ92_3552 | monooxygenase | 2,03772489 | 0,000107996 |
| HTZ92_1031 | hypothetical protein | 2,037397981 | 0,004975739 |
| HTZ92_2180 | transporter, major facilitator family protein | 2,036161996 | 0,000325214 |
| HTZ92_0137 | endonuclease | 2,035991238 | 4,91288E-05 |
| HTZ92_1375 | hypothetical protein | 2,024745158 | 0,001362478 |
| HTZ92_1083 | glycine zipper | 2,020887488 | 0,000246856 |
| HTZ92_0064 | efflux transporter, RND family | 2,01768528 | 0,001146098 |
| HTZ92_3290 | hypothetical protein | 2,01189483 | 0,003804048 |
| HTZ92_3285 | hypothetical protein | 2,011742033 | 0,020882312 |
| HTZ92_1019 | transporter | 2,010181752 | 0,003938653 |
| HTZ92_0694 | deoxyribodipyrimidine photo-lyase | 2,006925308 | 0,000416106 |
| HTZ92_3494 | hypothetical protein | 2,001308889 | 0,005115023 |
| HTZ92_0782 | ABC transporter | 2,001021401 | 0,000148835 |
| HTZ92_3600 | acetyl-coenzyme A synthetase | -2,000587443 | 2,25202E-05 |
| HTZ92_0727 | aldehyde dehydrogenase | -2,002853318 | 3,46465E-05 |
| HTZ92_2883 | protein-export protein SecB | -2,007441029 | 3,88481E-05 |
| HTZ92_0525 | carbamoyl-phosphate synthase large chain | -2,009965868 | 2,50696E-06 |
| HTZ92_1318 | N-acetyl-gamma-glutamyl-phosphate reductase | -2,010409711 | 7,84215E-07 |
| HTZ92_3538 | hypothetical protein | -2,010593695 | 2,25202E-05 |
| HTZ92_3230 | arginine-tRNA ligase | -2,015605171 | 3,73758E-05 |
| HTZ92_3296 | aromatic amino acid aminotransferase | -2,016358352 | 3,05988E-06 |
| HTZ92_1186 | 3-ketoacyl-ACP reductase | -2,020935142 | 2,367E-06 |
| HTZ92_1828 | taurine dioxygenase | -2,021151324 | 5,85646E-06 |
| HTZ92_0179 | putative lysine decarboxylase | -2,023034219 | 1,01651E-05 |
| HTZ92_1001 | sodium/glutamate symporter | -2,023545382 | 1,83092E-06 |
| HTZ92_3292 | AcnM | -2,023811404 | 6,43788E-05 |
| HTZ92_2760 | quinolinate synthetase | -2,027477565 | 1,94554E-07 |
| HTZ92_1185 | acetyl-CoA acetyltransferase | -2,028157588 | 6,22702E-06 |
| HTZ92_1780 | amino acid ABC transporter permease | -2,028276139 | 6,95821E-06 |
| HTZ92_1573 | diacetyl reductase | -2,031664545 | 0,001723408 |
| HTZ92_0530 | ribosomal RNA large subunit methyltransferase E | -2,032486076 | 7,01863E-06 |
| HTZ92_3238 | GMP synthetase | -2,034882616 | 4,09925E-07 |
| HTZ92_2789 | BolA-like protein | -2,035218686 | 3,07275E-05 |
| HTZ92_0275 | 4-hydroxythreonine-4-phosphate dehydrogenase | -2,040054837 | 4,29024E-07 |
| HTZ92_2743 | hypothetical protein | -2,041693196 | 0,000264994 |
| HTZ92_2643 | outer membrane lipoprotein LolB | -2,044788534 | 2,65986E-05 |
| HTZ92_2874 | MacA family efflux pump subunit | -2,046572002 | 5,18656E-06 |
| HTZ92_0507 | succinate dehydrogenase iron-sulfur subunit | -2,047018709 | 1,80028E-05 |
| HTZ92_0132 | Multidrug efflux transport protein | -2,048942214 | 1,23696E-06 |
| HTZ92_0066 | cation transporter | -2,052132652 | 1,13301E-05 |
| HTZ92_0049 | imidazoleglycerol-phosphate dehydratase | -2,052211105 | 6,8669E-07 |
| HTZ92_1543 | succinyl-CoA--3-ketoacid-CoA transferase | -2,060705497 | 1,26146E-06 |
| HTZ92_2797 | MarR family transcriptional regulator | -2,061915259 | 2,24443E-05 |
| HTZ92_0822 | acyl-CoA dehydrogenase | -2,062107835 | 7,01817E-06 |
| HTZ92_1700 | ATP-binding protein | -2,066278484 | 5,95156E-07 |
| HTZ92_3443 | AdeT, RND type efflux pump | -2,069162832 | 8,18948E-06 |
| HTZ92_2183 | ATP-dependent helicase HrpA | -2,069580452 | 1,82664E-08 |
| HTZ92_3489 | 4-hydroxy-tetrahydrodipicolinate synthase | -2,07238184 | 3,55562E-05 |
| HTZ92_0186 | acyl-CoA thioesterase | -2,076667048 | 3,97943E-06 |
| HTZ92_1980 | subunit of Phenylacetate-CoA oxygenase, phenylacetic acid degradation | -2,080292309 | 0,001355535 |
| HTZ92_0967 | nitroreductase | -2,082179881 | 3,23349E-06 |
| HTZ92_2198 | OmpA family | -2,082923135 | 0,00022549 |
| HTZ92_2787 | ATP phosphoribosyltransferase | -2,090502746 | 6,13952E-07 |
| HTZ92_0117 | tRNA (guanine-N(1)-)-methyltransferase | -2,10352626 | 6,21828E-05 |
| HTZ92_0171 | fructose-1,6-bisphosphatase | -2,106001193 | 3,90888E-06 |
| HTZ92_1293 | hypothetical protein | -2,109420005 | 0,000518119 |
| HTZ92_2710 | uracil phosphoribosyltransferase | -2,111468277 | 3,97431E-06 |
| HTZ92_2892 | 23S rRNA (adenine(2503)-C(2))-methyltransferase RlmN | -2,111903048 | 3,65212E-05 |
| HTZ92_3447 | anhydro-N-acetylmuramic acid kinase | -2,11872306 | 1,46901E-07 |
| HTZ92_0790 | hypothetical protein | -2,133444262 | 1,26374E-06 |
| HTZ92_0726 | 2,4-diaminobutyrate decarboxylase | -2,146303799 | 2,82848E-07 |
| HTZ92_0220 | 30S ribosomal protein S4 | -2,152652033 | 3,40046E-06 |
| HTZ92_2704 | Conserved TM helix | -2,157577847 | 2,48754E-05 |
| HTZ92_2895 | phosphatidylglycerophosphatase | -2,158695345 | 5,92016E-06 |
| HTZ92_0362 | hemolysin III | -2,166191598 | 3,02523E-05 |
| HTZ92_2418 | dioxygenase | -2,167248165 | 2,58368E-06 |
| HTZ92_1161 | mechanosensitive ion channel protein MscS | -2,167511745 | 2,10477E-06 |
| HTZ92_3609 | cation acetate symporter | -2,170276791 | 4,16938E-08 |
| HTZ92_3540 | hypothetical protein | -2,171533994 | 4,3167E-06 |
| HTZ92_3108 | DNA-directed RNA polymerase subunit beta | -2,176398632 | 2,42686E-08 |
| HTZ92_0052 | acetyl-CoA hydrolase | -2,185522392 | 5,16156E-07 |
| HTZ92_1521 | molybdenum ABC transporter substrate-binding protein | -2,202508893 | 6,91797E-07 |
| HTZ92_0966 | hypothetical protein | -2,205844823 | 1,59682E-05 |
| HTZ92_3028 | hypothetical protein | -2,226568943 | 2,37049E-05 |
| HTZ92_2767 | hypothetical protein | -2,230138145 | 1,57103E-06 |
| HTZ92_2516 | choline transporter | -2,239174055 | 1,3217E-07 |
| HTZ92_3189 | Endoribonuclease L-PSP | -2,243750907 | 6,24476E-05 |
| HTZ92_0054 | DNA-binding response regulator | -2,247187407 | 1,11094E-07 |
| HTZ92_0725 | diadenosine tetraphosphatase | -2,250973876 | 3,89598E-07 |
| HTZ92_0600 | ABC transporter | -2,264244248 | 2,73049E-08 |
| HTZ92_1139 | aldehyde dehydrogenase | -2,27700194 | 0,000235535 |
| HTZ92_2872 | lipoprotein | -2,279569438 | 6,22702E-06 |
| HTZ92_3010 | ABC transporter | -2,281559053 | 3,72493E-07 |
| HTZ92_0787 | ErfK/YbiS/YcfS/YnhG | -2,283529917 | 1,98568E-06 |
| HTZ92_2187 | hypothetical protein | -2,287997054 | 8,6871E-06 |
| HTZ92_2608 | thioredoxin reductase | -2,290591579 | 6,46047E-07 |
| HTZ92_3581 | dihydrolipoamide acetyltransferase | -2,292869534 | 6,27489E-07 |
| HTZ92_0744 | hypothetical protein | -2,294005232 | 1,97565E-06 |
| HTZ92_0944 | bifunctional protein | -2,324564379 | 4,33373E-09 |
| HTZ92_0605 | hydrolase | -2,325091649 | 1,94815E-08 |
| HTZ92_2591 | hypothetical protein | -2,339733197 | 4,62324E-07 |
| HTZ92_1572 | 2,3-butanediol dehydrogenase | -2,342860197 | 0,000251797 |
| HTZ92_3263 | 3-hydroxyisobutyrate dehydrogenase | -2,345414239 | 3,66456E-05 |
| HTZ92_0386 | GTP-binding protein TypA/BipA | -2,346887647 | 6,42414E-09 |
| HTZ92_2858 | aconitate hydratase | -2,349345205 | 5,95156E-07 |
| HTZ92_2298 | cholesterol oxidase | -2,351420192 | 1,60481E-08 |
| HTZ92_1063 | peptidase | -2,353028138 | 4,87437E-06 |
| HTZ92_0979 | glutamyl-tRNA amidotransferase | -2,359091953 | 5,58456E-05 |
| HTZ92_3110 | 50S ribosomal protein L10 | -2,359639823 | 4,39586E-07 |
| HTZ92_1593 | hypothetical protein | -2,360317192 | 0,000292426 |
| HTZ92_3260 | enoyl-CoA hydratase | -2,368788151 | 1,27308E-06 |
| HTZ92_1710 | fructose-bisphosphate aldolase | -2,37713116 | 5,96292E-07 |
| HTZ92_3039 | 30S ribosomal protein S15 | -2,380910942 | 2,29168E-05 |
| HTZ92_2875 | MacB family efflux pump subunit | -2,386321437 | 4,29024E-07 |
| HTZ92_0382 | RNA-splicing ligase RtcB | -2,39217852 | 6,23057E-08 |
| HTZ92_3580 | pyruvate dehydrogenase, E1 component | -2,395008736 | 1,76084E-06 |
| HTZ92_0647 | sulfate ABC transporter periplasmic substrate-binding protein | -2,396955549 | 1,0909E-05 |
| HTZ92_2416 | lon protease | -2,404629997 | 1,23995E-07 |
| HTZ92_1829 | taurine ABC transporter permease | -2,413105348 | 5,29316E-05 |
| HTZ92_2659 | metal-binding protein | -2,430793668 | 1,92105E-07 |
| HTZ92_0681 | glutamate 5-kinase | -2,436286241 | 2,33306E-08 |
| HTZ92_0095 | LemA family protein | -2,439483455 | 3,1747E-06 |
| HTZ92_0172 | DEAD/DEAH box helicase | -2,442000976 | 7,78707E-09 |
| HTZ92_0341 | stearoyl-CoA 9-desaturase | -2,449626857 | 2,73121E-09 |
| HTZ92_0659 | GTPase Era | -2,452182518 | 6,01879E-09 |
| HTZ92_3262 | AMP-binding protein | -2,455591637 | 3,13897E-06 |
| HTZ92_1321 | ATP-binding protease component | -2,468260805 | 2,36585E-07 |
| HTZ92_0128 | hypothetical protein | -2,470495628 | 7,77086E-06 |
| HTZ92_0258 | CDP-diacylglycerol--serine O-phosphatidyltransferase | -2,470682652 | 3,32551E-08 |
| HTZ92_1550 | aspartate ammonia-lyase | -2,473603214 | 1,04838E-06 |
| HTZ92_1574 | dihydrolipoamide dehydrogenase | -2,476707992 | 0,000134277 |
| HTZ92_3204 | peptide deformylase | -2,479163396 | 1,54851E-07 |
| HTZ92_2914 | trigger factor | -2,48508625 | 6,34371E-10 |
| HTZ92_2633 | ferredoxin | -2,491461019 | 5,64199E-09 |
| HTZ92_3063 | ribosome maturation factor RimP | -2,499973389 | 5,06874E-06 |
| HTZ92_3293 | methylcitrate synthase | -2,519270644 | 4,31818E-05 |
| HTZ92_2278 | carbon storage regulator | -2,523676893 | 2,58368E-06 |
| HTZ92_3411 | hypothetical protein | -2,525049576 | 0,000191165 |
| HTZ92_2590 | RnfH family ubiquitin | -2,525326498 | 1,07484E-10 |
| HTZ92_1284 | hypothetical protein | -2,529705797 | 3,50101E-06 |
| HTZ92_2763 | membrane transporter | -2,531605415 | 4,89742E-05 |
| HTZ92_0508 | 2-oxoglutarate dehydrogenase E1 component | -2,532262795 | 2,77007E-10 |
| HTZ92_0372 | porin | -2,535674263 | 8,96152E-09 |
| HTZ92_2208 | osmotically inducible protein C | -2,541562828 | 1,6864E-06 |
| HTZ92_2828 | glmZ(sRNA)-inactivating NTPase | -2,545819424 | 3,13217E-10 |
| HTZ92_3100 | signal peptide protein | -2,561230004 | 0,000138448 |
| HTZ92_3577 | UDP-3-O-[3-hydroxymyristoyl] N-acetylglucosamine deacetylase | -2,563100391 | 1,64515E-06 |
| HTZ92_0529 | RNA-binding protein | -2,563391455 | 2,89524E-05 |
| HTZ92_1241 | ornithine carbamoyltransferase | -2,565412531 | 5,29544E-10 |
| HTZ92_1295 | hypothetical protein | -2,570216597 | 5,29544E-10 |
| HTZ92_0965 | rhlB | -2,572112034 | 1,18895E-09 |
| HTZ92_0243 | tryptophan repressor-binding protein | -2,587367512 | 5,30587E-09 |
| HTZ92_3140 | DnaK suppressor protein | -2,592047612 | 7,18366E-07 |
| HTZ92_1075 | phosphoenolpyruvate synthase | -2,603236087 | 2,08959E-08 |
| HTZ92_3524 | SCP-2 sterol transfer family protein | -2,61349063 | 1,26146E-06 |
| HTZ92_2419 | signal peptide protein | -2,617845113 | 5,69681E-07 |
| HTZ92_1782 | ABC transporter substrate-binding protein | -2,634301724 | 1,32189E-06 |
| HTZ92_1310 | peptidase M16 | -2,634395107 | 1,82664E-08 |
| HTZ92_2881 | rhodanese | -2,636742666 | 2,57071E-08 |
| HTZ92_2214 | ATP phosphoribosyltransferase regulatory subunit | -2,649153235 | 1,09418E-10 |
| HTZ92_0811 | superoxide dismutase | -2,656879323 | 2,03372E-06 |
| HTZ92_0830 | ribosomal protein S2 | -2,65918726 | 3,7478E-08 |
| HTZ92_0962 | RNA polymerase factor sigma-32 | -2,673242951 | 4,07477E-09 |
| HTZ92_1422 | GntR family transcriptional regulator | -2,683732975 | 6,01879E-09 |
| HTZ92_0377 | hypothetical protein | -2,695198392 | 7,07181E-06 |
| HTZ92_2878 | Bax inhibitor 1 like | -2,698562484 | 3,62184E-08 |
| HTZ92_3184 | Integral membrane protein TerC family | -2,711438823 | 5,70077E-09 |
| HTZ92_1567 | sulfite exporter TauE/SafE | -2,732667042 | 3,44644E-09 |
| HTZ92_2189 | hypothetical protein | -2,734396915 | 2,74313E-08 |
| HTZ92_3036 | MerR family transcriptional regulator | -2,735630836 | 2,98808E-09 |
| HTZ92_2979 | protein chain initiation factor IF-1 | -2,736077695 | 5,06701E-09 |
| HTZ92_1838 | ubiquinol oxidase subunit II, cyanide insensitive | -2,741853936 | 1,32E-11 |
| HTZ92_0336 | acyl-CoA dehydrogenase | -2,751659514 | 5,7118E-10 |
| HTZ92_0380 | OmpA family | -2,765779258 | 8,67546E-08 |
| HTZ92_3261 | acyl-CoA dehydrogenase | -2,843491631 | 6,74326E-07 |
| HTZ92_0096 | TPM domain-containing protein | -2,854153435 | 4,49862E-10 |
| HTZ92_1051 | NLPA lipoprotein | -2,854327667 | 4,54801E-09 |
| HTZ92_3372 | hypothetical protein | -2,864441151 | 2,75E-11 |
| HTZ92_2612 | metal-dependent hydrolase | -2,885458464 | 1,74E-11 |
| HTZ92_2769 | D-ribulose-5-phosphate 3-epimerase | -2,923574281 | 9,74584E-10 |
| HTZ92_3481 | hypothetical protein | -2,928564538 | 5,29544E-07 |
| HTZ92_1830 | taurine ABC transporter ATP-binding protein | -2,9375565 | 3,0203E-09 |
| HTZ92_1568 | NAD(FAD)-dependent dehydrogenase | -2,964676828 | 4,33E-12 |
| HTZ92_2088 | LPS export ABC transporter periplasmic protein LptC | -2,975311376 | 5,91E-13 |
| HTZ92_0249 | ATP-binding protein | -2,990681605 | 3,17E-15 |
| HTZ92_3539 | hypothetical protein | -2,998171149 | 3,90167E-06 |
| HTZ92_2069 | hypothetical protein | -2,999191333 | 2,34E-12 |
| HTZ92_1886 | hydroperoxidase II | -3,000431358 | 7,7994E-10 |
| HTZ92_1127 | tRNA dimethylallyltransferase | -3,016889064 | 3,29649E-08 |
| HTZ92_2634 | phosphopantetheine adenylyltransferase | -3,041693455 | 8,77E-12 |
| HTZ92_0557 | ABC transporter substrate-binding protein | -3,04872041 | 3,68675E-10 |
| HTZ92_1619 | haloacid dehalogenase | -3,053875555 | 2,61E-14 |
| HTZ92_2671 | trehalose-6-phosphate synthase | -3,088900027 | 1,41306E-06 |
| HTZ92_1007 | CsuA/B | -3,108342657 | 9,37E-15 |
| HTZ92_1068 | 30S ribosomal protein S6 | -3,123759347 | 2,21E-12 |
| HTZ92_3025 | magnesium transporter | -3,131372611 | 1,84E-11 |
| HTZ92_1683 | 30S ribosomal protein S1 | -3,136211179 | 1,53E-16 |
| HTZ92_3383 | SDR family oxidoreductase | -3,177674253 | 1,33E-12 |
| HTZ92_2400 | arginine exporter protein ArgO | -3,185788703 | 1,49717E-08 |
| HTZ92_1569 | Zn-dependent hydrolase | -3,331680481 | 3,02652E-09 |
| HTZ92_3264 | methylmalonate-semialdehyde dehydrogenase (acylating) | -3,359271957 | 4,49862E-10 |
| HTZ92_0792 | class C beta-lactamase | -3,424110042 | 1,09E-16 |
| HTZ92_0105 | DNA-directed RNA polymerase subunit omega | -3,461158735 | 2,9994E-08 |
| HTZ92_2960 | hypothetical protein | -3,464471324 | 1,84E-11 |
| HTZ92_0060 | Sel1 repeat protein | -3,533451049 | 9,50E-18 |
| HTZ92_1995 | hemolysin | -3,574793629 | 8,7644E-09 |
| HTZ92_2673 | bacterioferritin | -4,053864419 | 4,82E-15 |
| HTZ92_3591 | Putative general bacterial porin | -4,147022962 | 9,30E-25 |
| HTZ92_2157 | cold-shock protein | -4,33042974 | 1,87807E-10 |

**Tab. S2. pH of culture media and timepoint of VBNC state entry after growth on different carbon sources.**

| Carbon source | Timepoint of VBNC state induction (days) | pH of the stationary culture |
| --- | --- | --- |
| succinate | 4 ± 1 | 9.5 |
| glutamate | 7 ± 2 | 9.5 |
| histidine | 18 ± 2 | 8 |
| alanine | 25 ± 2 | 8 |
| xylose | 30 ± 4 | 7 |
| arabinose | 45 ± 5 | 7 |
| ethanol | 63 ± 3 | 7 |


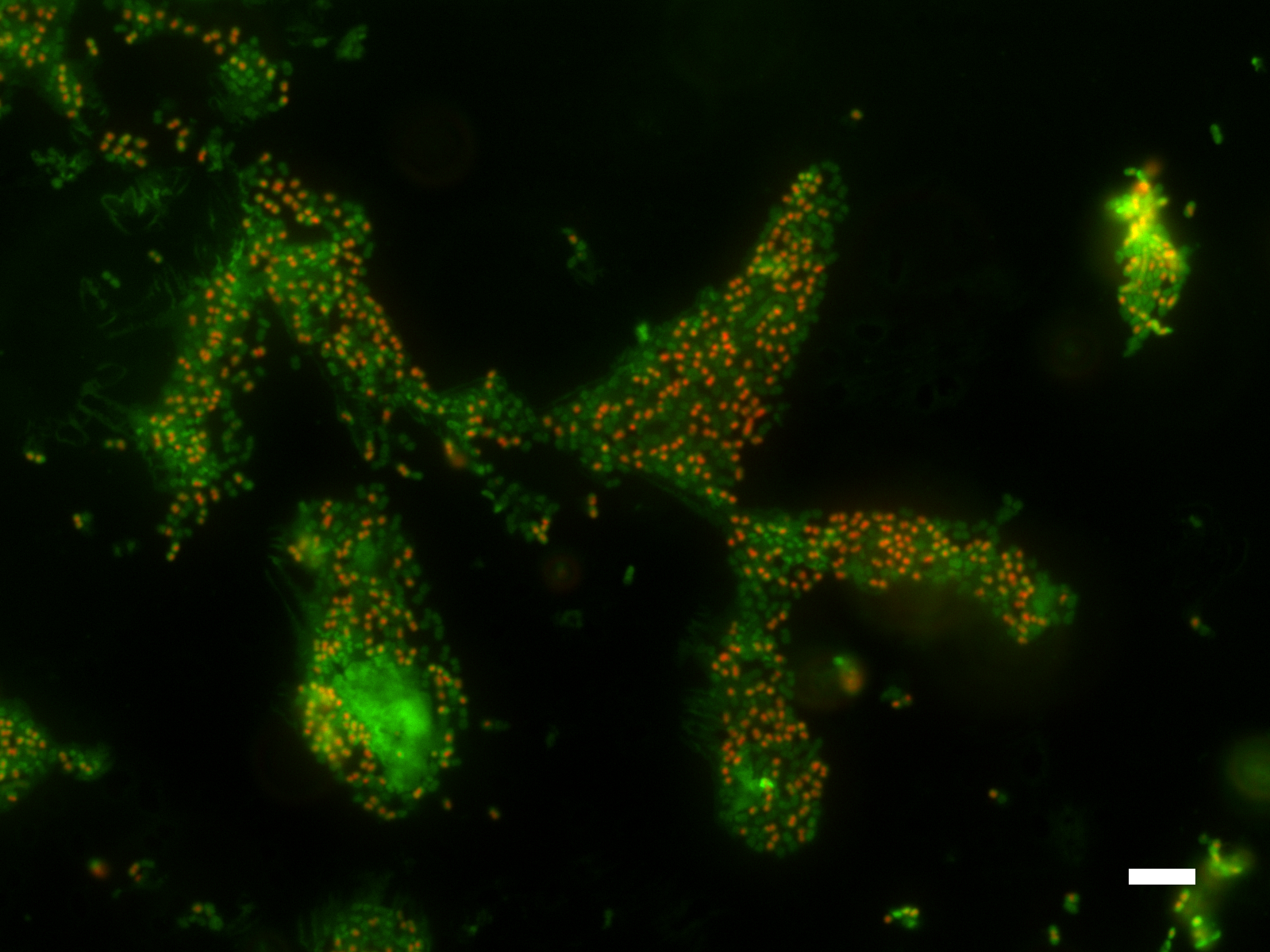


**FIG S1 Viability of *A. baumannii* as determined by LIVE/DEAD staining and fluorescence microscopy.** Cells were grown under high salt conditions to four days PSP (T4) and stained with Syto9 and PI (LIVE/DEAD^TM^ BacLight^TM^ Bacterial Viability Kit) according to manufacturer’s instructions. Stained cells were qualitatively assessed by fluorescence microscopy (Zeiss Axio Imager M1) using a 63x objective. Green-fluorescing cells are considered as viable, while red-fluorescing cells are considered as dead. The white bar indicates the 10 µm size standard.


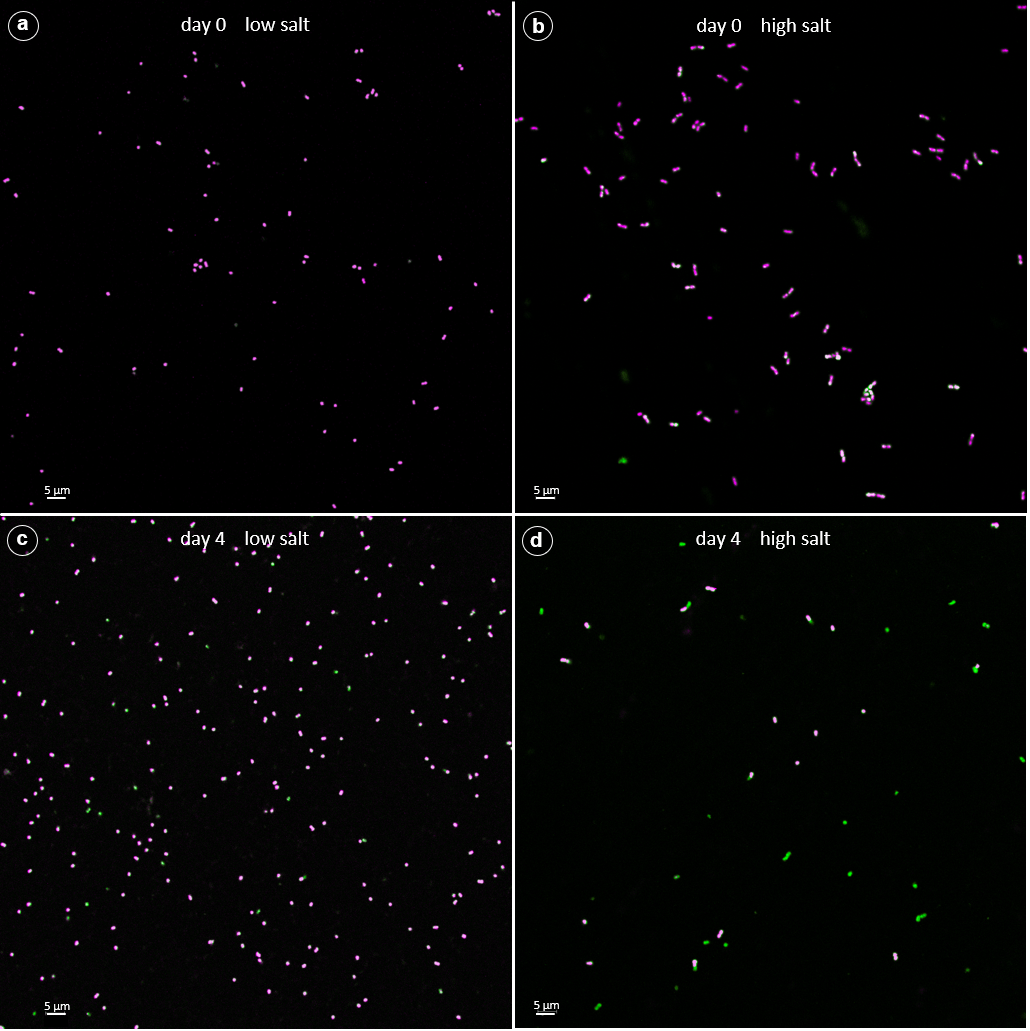


**FIG S2 Visualization of bacterial respiratory activity using CTC staining.** Confocal laser scanning microscopy of *A. baumannii* (ATCC 19606^T^) after reaching post stationary phase (T0) (a, b) and after 4 days PSP (T4) (c, d) cultivation in low salt media (a, c) and high salt media (b, d). Respiratory activity (CTC stain): magenta, dsDNA (DAPI): green.


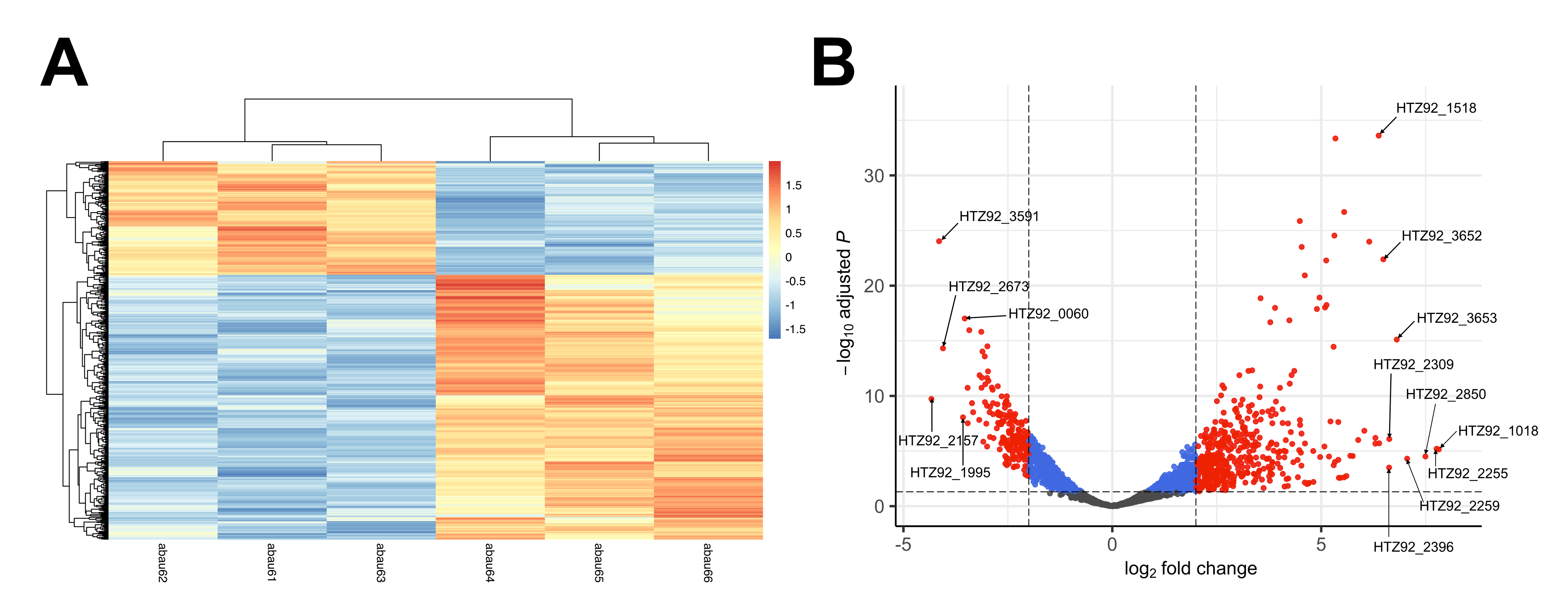


**FIG S3 Overview of differentially regulated genes identified in VBNC state cells compared to stationary cells under high salt stress. (A)** Expression heat map of 618 differentially regulated genes showing higher (red) and lower levels of expression (blue) **(B)** Volcano plot of differentially regulated genes showing 186 downregulated and 432 upregulated genes (red dots) with a log_2_ fold change ≥ 2 and ≤ -2. Blue dots indicate an expression level within a log_2_ fold change of 2 to -2, while grey dots visualize genes without significant differential expression. Locus tags of highly down- or upregulated genes are denoted.


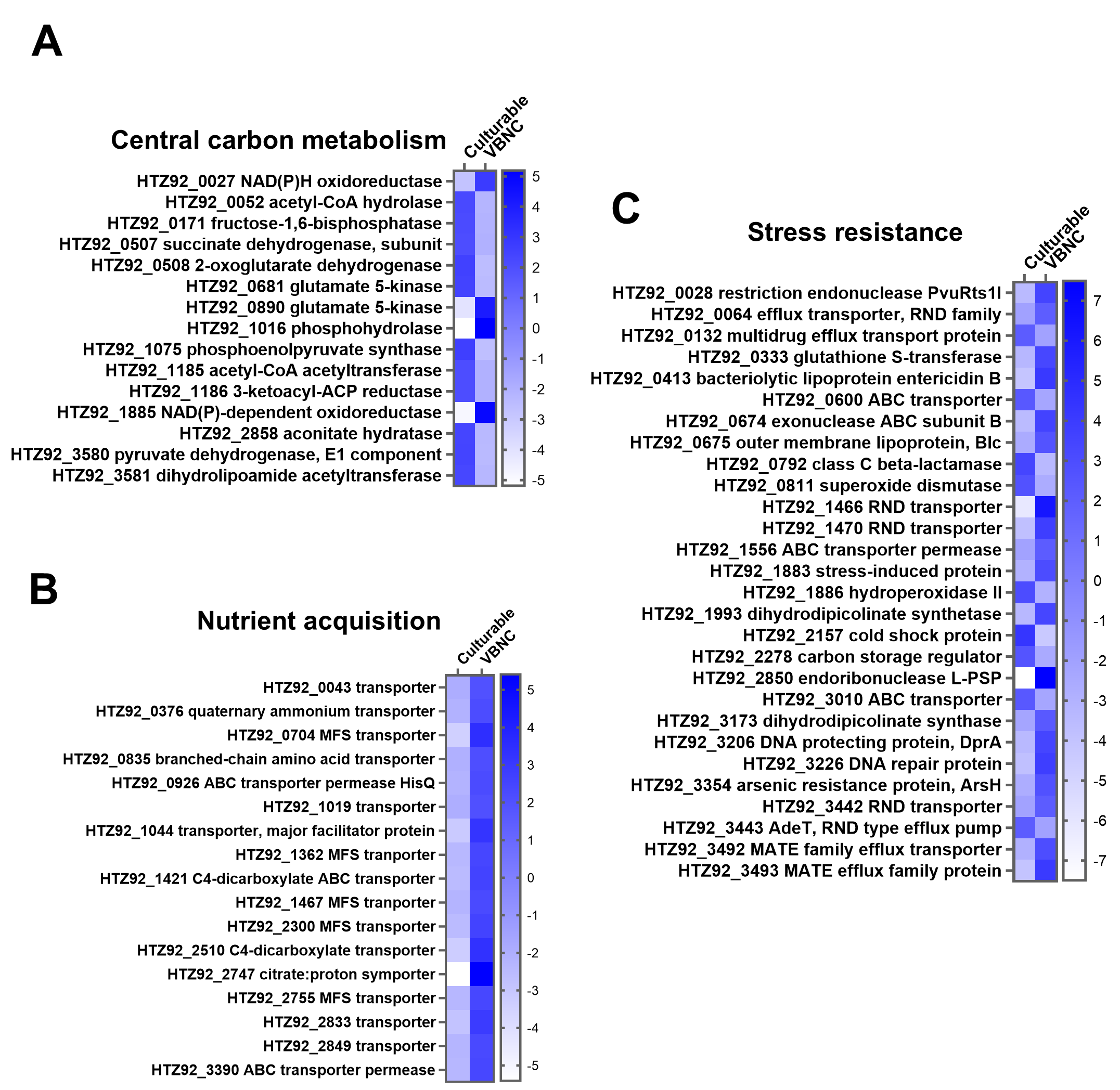


**FIG S4** **Top ranked DEGs in the categories central carbon metabolism (A), nutrient acquisition (B) and stress resistance (C).** Heatmaps of up- and downregulated genes (p value ≤ 0.05 and a log_2_FC ≥ 2) involved in central carbon metabolism, nutrient acquisition or stress resistance. The differences are visualized with a color code represented by the color bar on the right. Figures were generated with GraphPad Prism 9.


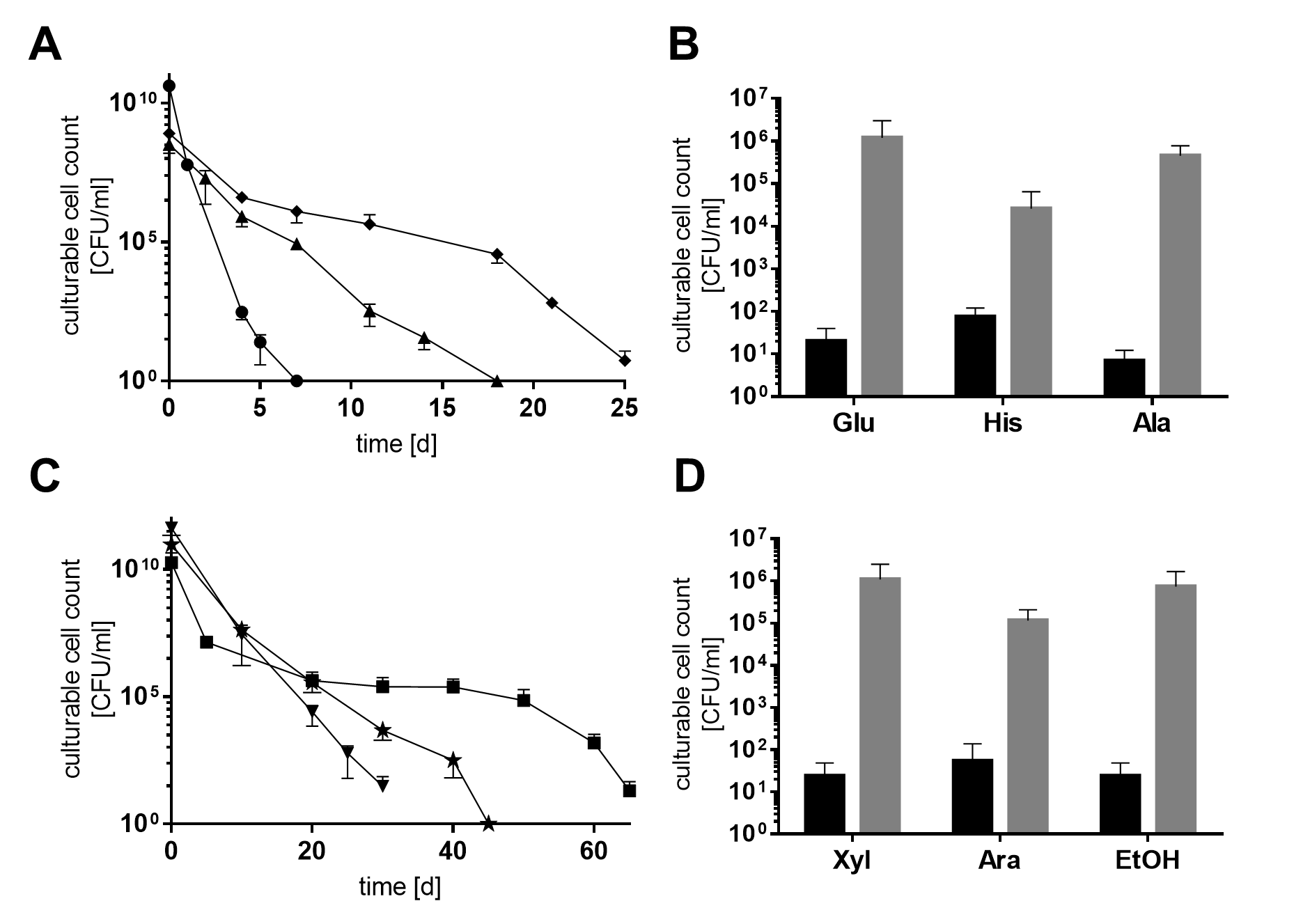


FIG S5 VBNC state entry of salt-stressed cells is carbon source-dependent. *A. baumannii* ATCC 19606 was grown in mineral medium on different carbon sources (● = 20 mM glutamate, ▲ = 10 mM histidine, ♦ = 20 mM alanine; C: ▼ = 20 mM xylose, ★ = 20 mM arabinose, ■ = 20 mM EtOH) in the presence of 300 mM NaCl. At timepoints indicated culturability was addressed by plating samples on LB agar plates (A+B). Once culturability dropped below 100 CFU/ml, a resuscitation assay was performed (C+D). The stressor was removed by diluting cells 1:10 in sterile PBS. Samples were plated directly after inoculation (black bars) and after incubating the cells for 2 days at 37°C (grey bars). Error bars denote the standard deviation of the mean derived from at least three independent biological replicates.


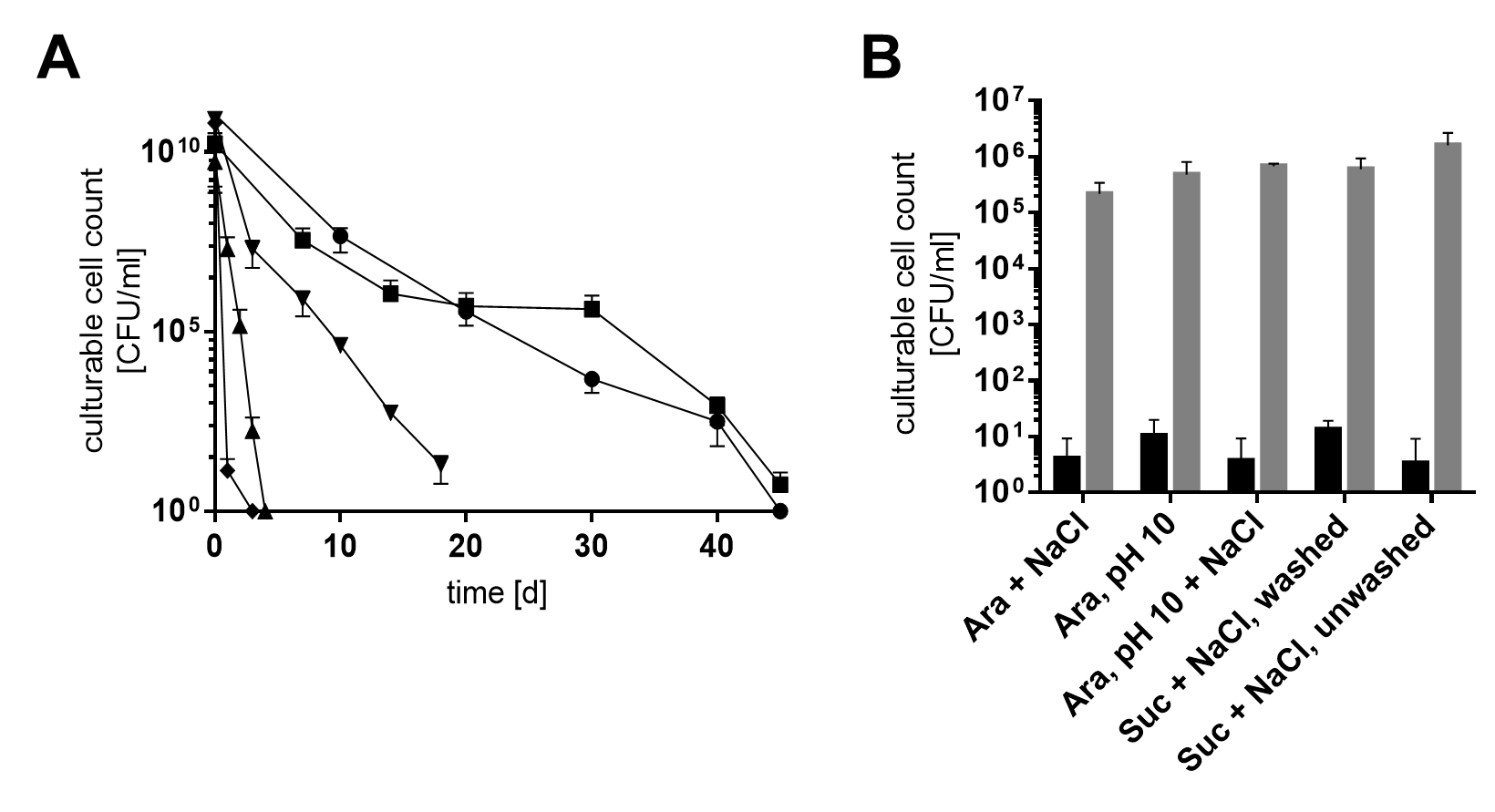


**FIG S6 Combination of pH and salt stress accelerates VBNC state entry.** Growth of *A. baumannii* in mineral medium on 20 mM succinate and 300 mM NaCl (▲) shifted the pH of the medium to pH 10. To remove this additional stress, succinate-grown cells were washed in sterile saline prior to subjecting them to 300 mM NaCl (■). Since utilization of arabinose did not change the pH, cells grown on arabinose in the presence of either 300 mM NaCl (●), pH 10 (▼) or both stressors (♦), served as controls. At timepoints indicated culturability was addressed by plating samples on LB agar plates **(A)**. Once culturability dropped below 100 CFU/ml, a resuscitation assay was performed **(B)**. The stressor was removed by diluting cells 1:10 in sterile PBS. Samples were plated directly after inoculation (black bars) and after incubating the cells for 2 days at 37°C (grey bars). Error bars denote the standard deviation of the mean derived from at least three independent biological replicates.


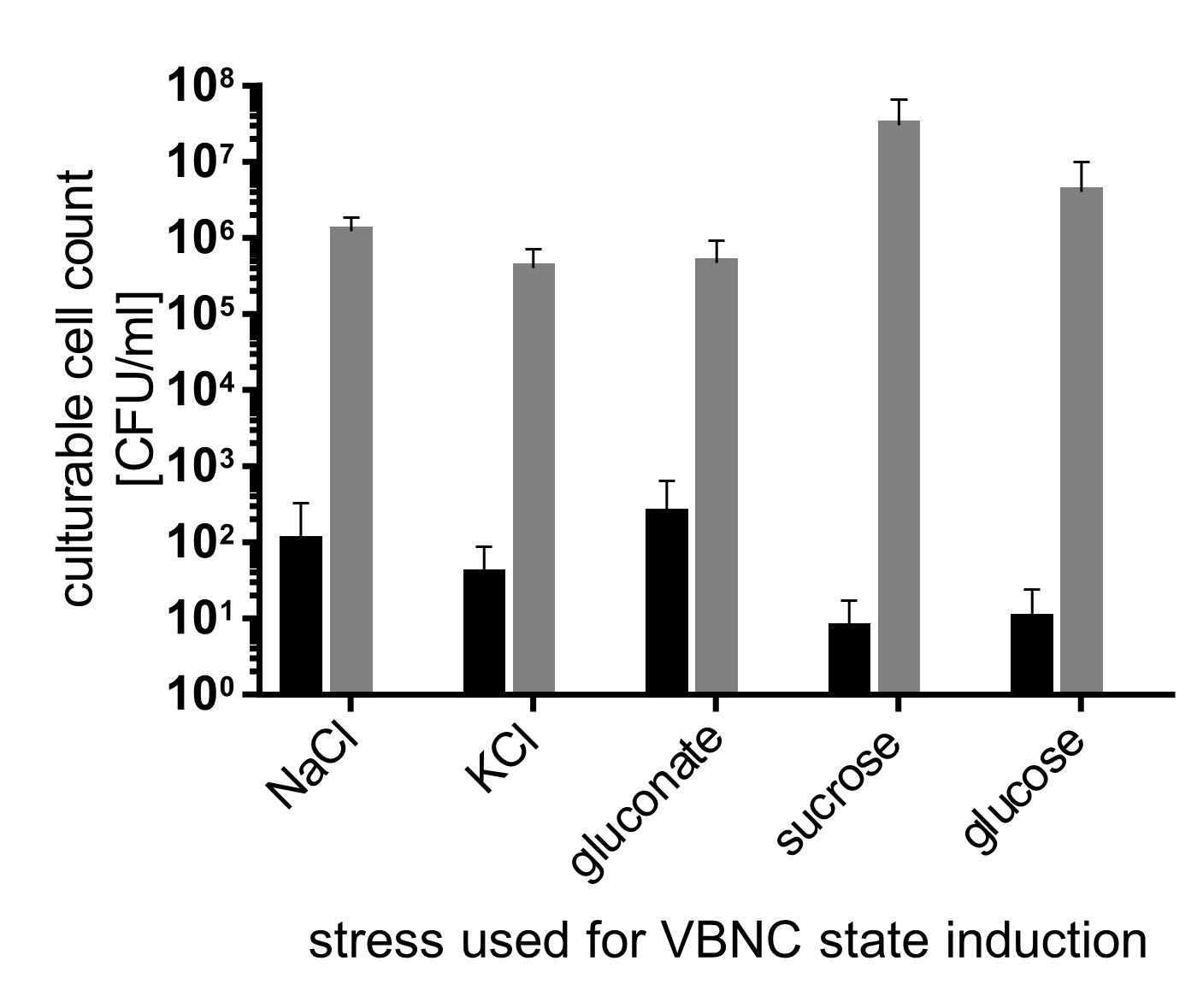


**FIG S7** **Resuscitation of VBNC cells induced by different osmolytes*.*** The VBNC state was induced by the presence of either 300 mM NaCl, 300 mM KCl, 300 mM Na^+^-gluconate, 600 mM sucrose or 600 mM glucose. Culturability was addressed by plating samples on LB agar plates. Once culturability dropped below 100 CFU/ml, a resuscitation assay was performed. The stressor was removed by diluting cells 1:10 in sterile PBS. CFU/ml were determined directly after inoculation (black bars) and after incubating the cells for 2 days at 37°C (grey bars). Error bars denote the standard deviation of the mean derived from at least three independent biological replicates.


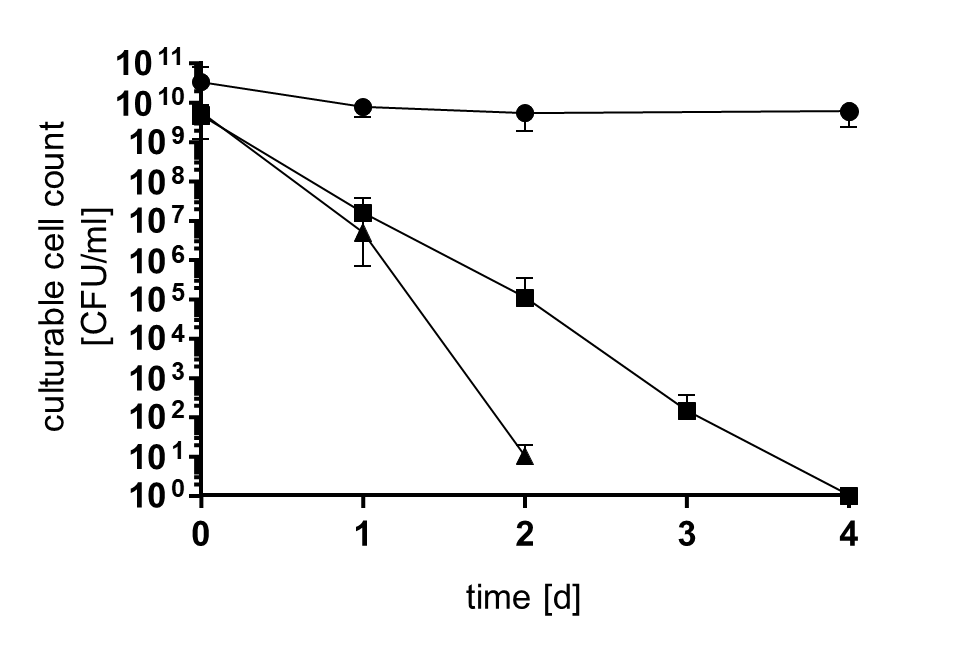


FIG S8 Salt stress in combination with temperature stress modulates VBNC state entry. Cells were grown in mineral medium containing 20 mM succinate and 300 mM NaCl to stationary growth phase before the culture was subjected to 4°C (●), 42°C (▲) and 37°C (■, reference). The culturable cell count was determined at indicated timepoints by plating samples on LB agar plates. Error bars denote the standard deviation of the mean derived from at least three independent biological replicates.


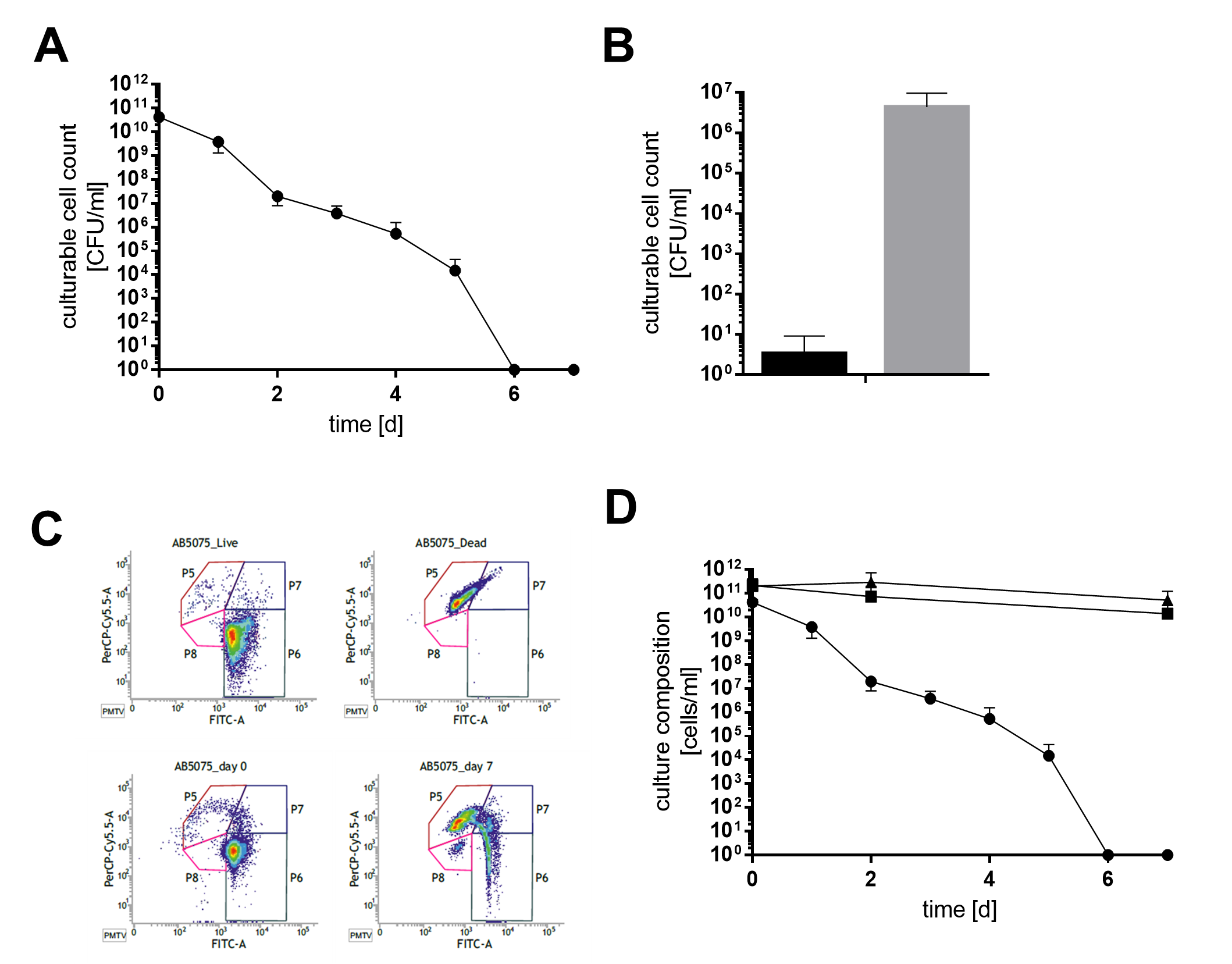


**FIG S9** **Survival of *A. baumannii* AB5075 under salt stress as determined by LIVE/DEAD staining.** *A. baumannii* AB5075 was grown in minimal medium with 20 mM succinate and 300 mM NaCl to stationary growth phase (timepoint 0) and for further 7 days. At timepoints indicated, the culturable cell count (●) was determined by serially diluting the culture and plating samples onto LB agar plates **(A)**. Once culturability dropped below 100 CFU/ml, a resuscitation assay was performed. The stressor was removed by diluting cells 1:10 in sterile PBS. CFU/ml were determined directly after inoculation (black bars) and after incubating the cells for 2 days at 37°C (grey bars, **B**). Additionally, at timepoints indicated, samples of the culture were stained with Syto9 and PI (LIVE/DEAD^TM^ BacLight^TM^ Bacterial Viability Kit) according to manufacturer’s instructions and analyzed with flow cytometry. Representative density plots from at least three independent biological replicates are shown **(C)**. The viable cell count (■) is defined as the sum of population 6 and 7, while the total cell count (▲) is defined as the sum of population 5 to 8 and quantified using the kit’s microsphere standard **(D)**.


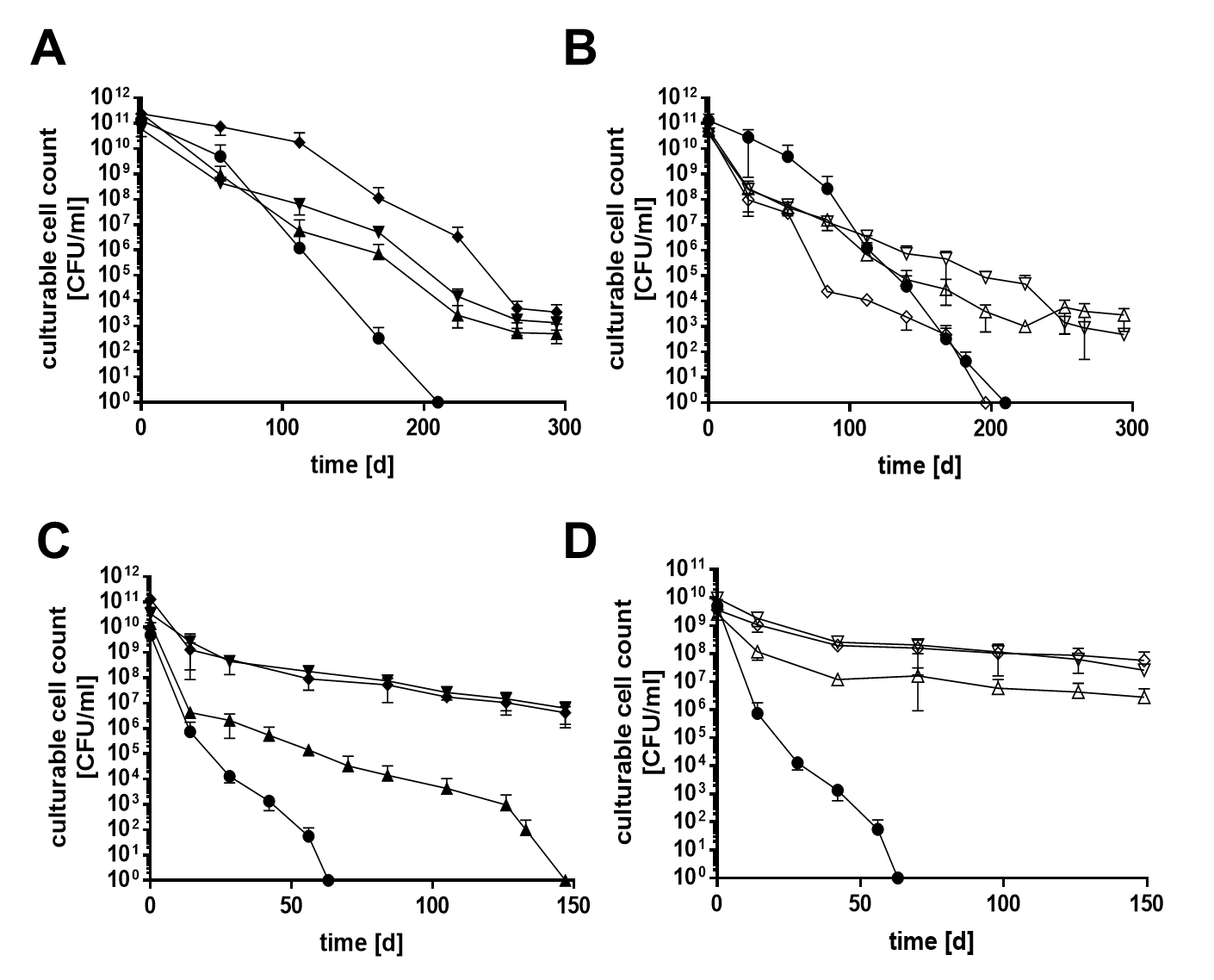


**FIG S10** **Culturability of other *A. baumannii* strains and clinical isolates under cold stress and desiccation.** Cells of the wild type (●), AB5075 (▼), AYE-T (♦), ACICU (▲), CS36 (♢), CS121 (∇) and SC1947 (∆) were analyzed regarding their persistence under cold stress **(A+B)** and desiccation **(C+D)**. Cultivation conditions were described in material and methods. Culturability was addressed by plating samples on LB agar plates at timepoints given. Error bars denote the standard deviation of the mean derived from at least three independent biological replicates.
